# Molecular Evolution and Expansion of the KUP Family in the Allopolyploid Cotton Species *Gossypium hirsutum* and *Gossypium barbadense*

**DOI:** 10.3389/fpls.2020.545042

**Published:** 2020-09-30

**Authors:** Kai Fan, Zhijun Mao, Jiaxin Zheng, Yunrui Chen, Zhaowei Li, Weiwei Lin, Yongqiang Zhang, Jinwen Huang, Wenxiong Lin

**Affiliations:** ^1^Key Laboratory of Ministry of Education for Genetics, Breeding and Multiple Utilization of Crops, College of Agriculture, Fujian Agriculture and Forestry University, Fuzhou, China; ^2^Fujian Provincial Key Laboratory of Agroecological Processing and Safety Monitoring, College of Life Sciences, Fujian Agriculture and Forestry University, Fuzhou, China; ^3^Key Laboratory of Crop Ecology and Molecular Physiology (Fujian Agriculture and Forestry University), Fujian Province University, Fuzhou, China

**Keywords:** cotton, KUP family, polyploidization, molecular evolution, expansion

## Abstract

The comprehensive analysis of gene family evolution will elucidate the origin and evolution of gene families. The K+ uptake (KUP) gene family plays important roles in K+ uptake and transport, plant growth and development, and abiotic stress responses. However, the current understanding of the KUP family in cotton is limited. In this study, 51 and 53 KUPs were identified in *Gossypium barbadense* and *Gossypium hirsutum*, respectively. These KUPs were divided into five KUP subfamilies, with subfamily 2 containing three groups. Different subfamilies had different member numbers, conserved motifs, gene structures, regulatory elements, and gene expansion and loss rates. A paleohexaploidization event caused the expansion of GhKUP and GbKUP in cotton, and duplication events in *G. hirsutum* and *G. barbadense* have happened in a common ancestor of *Gossypium*. Meanwhile, the KUP members of the two allopolyploid subgenomes of *G. hirsutum* and *G. barbadense* exhibited unequal gene proportions, gene structural diversity, uneven chromosomal distributions, asymmetric expansion rates, and biased gene loss rates. In addition, the KUP families of *G. hirsutum* and *G. barbadense* displayed evolutionary conservation and divergence. Taken together, these results illustrated the molecular evolution and expansion of the KUP family in allopolyploid cotton species.

## Introduction

Cotton (*Gossypium*) is a globally important natural source of fibers and is a good model for studying genome evolution and polyploidization in the plant kingdom. *Gossypium* has experienced two major whole-genome duplication events: a paleohexaploidization event and a cotton-specific decaploidy event (Paterson et al., 2012; Wang et al., 2016; Hu et al., 2019). The genus *Gossypium* consists of approximately 45 diploid (2n = 2x = 26) and five allopolyploid (2n = 4x = 52) species (Wendel and Albert, 1992). Diploid cotton species include eight genome groups (A–G and K genomes). Diploid cotton species, such as the A-genome species *Gossypium arboreum* and the D-genome species *Gossypium raimondii*, diverged from a common eudicot ancestor belonging to the *Gossypium* genus approximately 5–10 million years ago (MYA). Approximately 1–2 MYA, allopolyploid species, including the two widely cultivated species *Gossypium hirsutum* and *Gossypium barbadense*, originated from the transoceanic hybridization of the A and D genome species. Then, allopolyploid species independently evolved in diverse environments (Wendel, 1989; Paterson et al., 2012; Wang et al., 2017). *G. hirsutum* and *G. barbadense* have different morphologies and economic traits (Hu et al., 2019; Wang M. et al., 2019). Physiological and phenotypic divergences are related to the metabolism of mineral nutrients in cotton. Many mineral elements, such as phosphorus and calcium, play critical roles in regulating numerous cotton biological processes (Tang et al., 2014; Santos et al., 2019).

In plants, K+ is an essential macronutrient and controls many key biological processes, such as root growth and development (Sustr et al., 2019), leaf senescence (Wang et al., 2012), and fruit quality (Hartz et al., 2005). K+ can influence cotton yield and quality (Pettigrew, 2008). For example, the cell expansion of cotton fibers can be regulated by turgor pressure under the mediation of K+ concentration (Dhindsa et al., 1975). Plants absorb and transport K+ through K+ channels or transporters (Véry et al., 2014). K+ transporters in plants can be divided into the KT/HAK/K+ uptake (KUP), Trk/HKT, CHX, and KEA families (Mäser et al., 2001; Gupta et al., 2008). The KUP family is the largest K+ transporter family (Kim et al., 1998; Alemán et al., 2014; Han et al., 2016). It can regulate plant growth and developmental processes, including root hair (Ahn et al., 2004) and shoot cell expansion (Elumalai et al., 2002), and is associated with salt stress and osmotic regulation (Osakabe et al., 2013; Chen et al., 2015).

Members of the KUP family have been found in fungi, bacteria, and plants (Rodríguez-Navarro, 2000). A KUP member in plants was first identified in *Arabidopsis* (Quintero and Blatt, 1997). Since then, many KUP members have been reported in various flowering plants, such as *Oryza sativa* (Gupta et al., 2008), *Zea mays* (Zhang et al., 2012), *Triticum aestivum* (Cheng et al., 2018), *Solanum lycopersicum* (Hyun et al., 2014), *Populus trichocarpa* (He et al., 2012), *Prunus persica* (Song et al., 2015), *Cicer arietinum* (Azeem et al., 2018), *Nicotiana tabacum* (Song et al., 2019), and *Manihot esculenta* (Ou et al., 2018). Some KUP genes have been reported in cotton (Wang et al., 2018; Wang Y. et al., 2019). *G. hirsutum* K+ transporter 2 (GhKT2) may be related to K+ acquisition, transport, and distribution (Wang et al., 2018), and *G. hirsutum* high-affinity K+ transporter 5a (GhHAK5a) is essential for the shoot regulation of root K+ uptake under potassium deficiency (Wang Y. et al., 2019). However, a genome-wide systematic analysis of the KUP family in cotton does not exist.

The complete genome sequences of *G. hirsutum*, *G. barbadense*, and their putative diploid donor species *G. arboreum* and *G. raimondii* provide ideal opportunities for investigating the evolutionary and functional genomics of cotton (Du et al., 2018; Hu et al., 2019; Udall et al., 2019). A comprehensive investigation of molecular evolution is urgently needed to elucidate the complex evolutionary history of KUP proteins after cotton polyploidization. The comprehensive evolutionary history of the KUP family can unravel selection and accelerate the molecular breeding of cotton. In the present study, the KUP members of *Arabidopsis thaliana*, *O. sativa*, *Vitis vinifera*, *Theobroma cacao*, *Bombax ceiba*, *Corchorus capsulari*, *Corchorus olitorius*, *Herrania umbratica*, *Durio zibethinus*, *Gossypioides kirkii*, *Gossypium austral*, *Gossypium turneri*, *G. raimondii*, *G. arboretum*, *G. hirsutum*, and *G. barbadense* were subjected to genome-wide and comparative genomic analyses to reveal the molecular evolutionary history of the gene family, including the asymmetric evolution of subfamilies, the unequal evolution of subgenomes, and the conserved and divergent evolution of the two cultivated allopolyploid species.

## Materials and Methods

### Sequence Retrieval and Phylogenetic Analysis

The genome sequences of *A. thaliana*, *O. sativa*, *V. vinifera*, *T. cacao*, *B. ceiba*, *C. capsulari*, *C. olitorius*, *H.umbratica*, *D. zibethinus*, *G. kirkii*, *G. austral*, *G. turneri*, *G. raimondii*, *G. arboretum*, *G. hirsutum*, and *G. barbadense* were downloaded from publically available databases (**Table S1**). Hmmsearch in HMMER 3.0 program was conducted to identify the KUP members of the above-mentioned species by using the KUP domain (Pfam ID: PF02705) as the query (Finn et al., 2011). Then, the conserved KUP domains of the candidate KUP sequences were further confirmed by utilizing the CDD program with default settings (https://www.ncbi.nlm.nih.gov) (Marchler-Bauer et al., 2015). Finally, all of the KUP members with KUP domains (Pfam ID: PF02705) were retained for further analysis. The KUP members of *T. cacao*, *G. raimondii*, *G. arboretum*, *G. hirsutum*, and *G. barbadense* were designated as TcKUP, GrKUP, GaKUP, GhKUP, and GbKUP, respectively.

All of the KUP sequences were aligned by using MAFFT (Katoh and Standley, 2013). Parameters, except for globalpair and maxiterate, were set with the default settings. Maxiterate was set to 1,000. A phylogenetic tree was inferred *via* maximum likelihood (ML) by using IQ-tree (Kalyaanamoorthy et al., 2017). The best model was selected by applying the ModelFinder program, and the bootstrap value was set to 1,000 (Kalyaanamoorthy et al., 2017). All trees were visualized with MEGA software (Kumar et al., 2018).

### Structural and Promoter Analysis

The conserved motifs of the KUP family in *G. hirsutum*, *G. barbadense*, *G. arboretum*, and *G. raimondii* were identified with MEME (http://meme-suite.org/tools/meme) (Bailey et al., 2009). Parameters were set on the basis of values that were previously used to analyze putative motifs (Fan et al., 2019; Fan et al., 2020). The identified motifs were further annotated by using CDD (Marchler-Bauer et al., 2015). The structural and chromosomal location information of all the *KUP* genes in cotton was downloaded from CottonGen (https://www.cottongen.org/). The gene structures and chromosomal distribution images of *GhKUP*s, *GbKUP*s, *GaKUP*s, and *GrKUP*s were visualized with TBtools (Chen et al., 2020).

The 1000-bp upstream sequences of *GhKUP*s and *GbKUP*s were extracted from CottonGen. Potential *cis*-elements in the *GhKUP* and *GbKUP* promoters were identified with PlantCARE database (http://bioinformatics.psb.ugent.be/webtools/plantcare/html/). Stress-responsive regulatory elements, including the ABA-responsive element (ABRE), gibberellin-responsive element (GARE), defense and stress responsiveness (DSR), low-temperature-responsive element (LTR), and MYB-binding site (MBS), were visualized by using TBtools (Chen et al., 2020).

### Identification of Syntenic Blocks and Orthologous Gene Pairs

Syntenic blocks in *G. hirsutum*, *G. barbadense*, *G. arboretum*, and *G. raimondii* were identified with MCScan (Python version) (https://github.com/tanghaibao/jcvi/wiki/MCscan-[Python-version]). Each block contained at least five genes. Circos was utilized to visualize syntenic relationships (Krzywinski et al., 2009). Moreover, the orthologous gene pairs of four cotton species were searched on the basis of syntenic block and phylogenetic tree results. In addition, the nonsynonymous distance (Ka), synonymous distance (Ks), and their ratio (Ka/Ks) were estimated by using TBtools software with the Nei–Gojobori model (Nei and Gojobori, 1986; Chen et al., 2020). The density plots of Ks were analyzed and visualized by using ggplot2 package in RStudio (Studio, 2012; Wickham et al., 2016). Statistical analyses were performed in RStudio (Studio, 2012). In accordance with the neutral substitution rate of cotton (*r* = 2.6 × 10^−9^), the estimated divergence times were calculated by using the formula t = Ks/2*r* (Hu et al., 2019).

### Expression Analysis

*G. hirsutum* acc. TM-1 and *G. barbadense* acc. Hai7124 were used for expression analysis. Roots, stems, and leaves were harvested from 4-week-old seedlings grown in a greenhouse. For abiotic stress treatments, four-week-old seedlings were subjected to drought stress (20% PEG6000), salt stress (250 mM NaCl), and ABA treatment (100 mM ABA) in Hoagland’s solution. The third true leaves were collected at 0, 6, 12, and 24 h after treatment. Three biological replications were assayed. All collected samples were quick-frozen in liquid nitrogen and then stored at −80°C.

Total RNA was extracted from frozen samples with the RNAprep pure Plant Kit (TIANDZ, China), and first-strand cDNA was synthesized from DNase-treated RNA with a PrimerScript 1st Strand cDNA synthesis kit (TaKaRa, Dalian, China). Gene-specific primers for quantitative real-time PCR (qRT-PCR) analysis were designed and synthesized (**Table S2**). BLAST searches against the *G. hirsutum* and *G. barbadense* genomes were performed to confirm the primers’ specificity. qRT-PCR was run on a CFX96 Realtime System (BioRad) with SYBR premix Extaq (TaKaRa, Dalian, China). An annealing temperature of 60°C and 40 cycles were set for all qRT-PCR reactions. The cotton *EF1α* gene was selected as the endogenous control to normalize expression data. Relative expression levels were calculated in accordance with the comparative cycle threshold method. All reactions were conducted with three biological replications. The expression levels of *GhKUP*s and *GbKUP*s were clustered by using ComplexHeatmap package in RStudio (Studio, 2012; Gu et al., 2016). Statistical analyses were performed in RStudio (Studio, 2012). The Pearson correlation coefficients (PCCs) of the expression data were calculated and visualized by using ggcorrplot package in RStudio (Studio, 2012; Kassambara, 2018).

## Results

### Identification and Phylogenetic Analysis of KUPs in Malvaceae, *Arabidopsis*, Grape, and Rice

**Figure 1:**
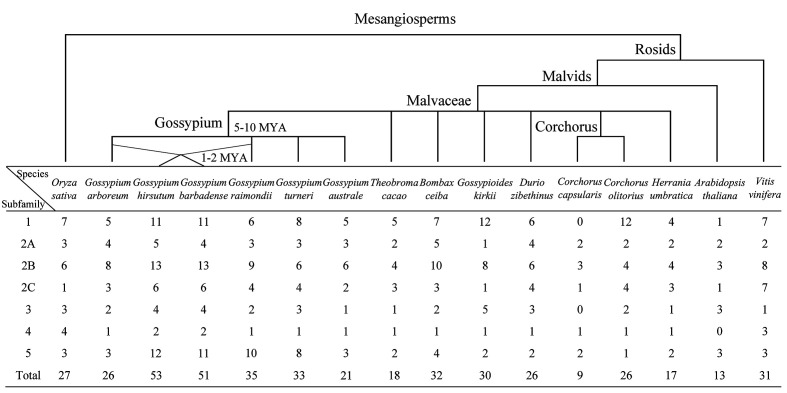
Distribution of KUP members in 13 Malvaceae species, *Arabidopsis*, grape, and rice. The upper branch displays the simple phylogenetic relationships of the 16 species. The bottom table represents the number of KUP genes in each KUP subfamily.

**Figure 2:**
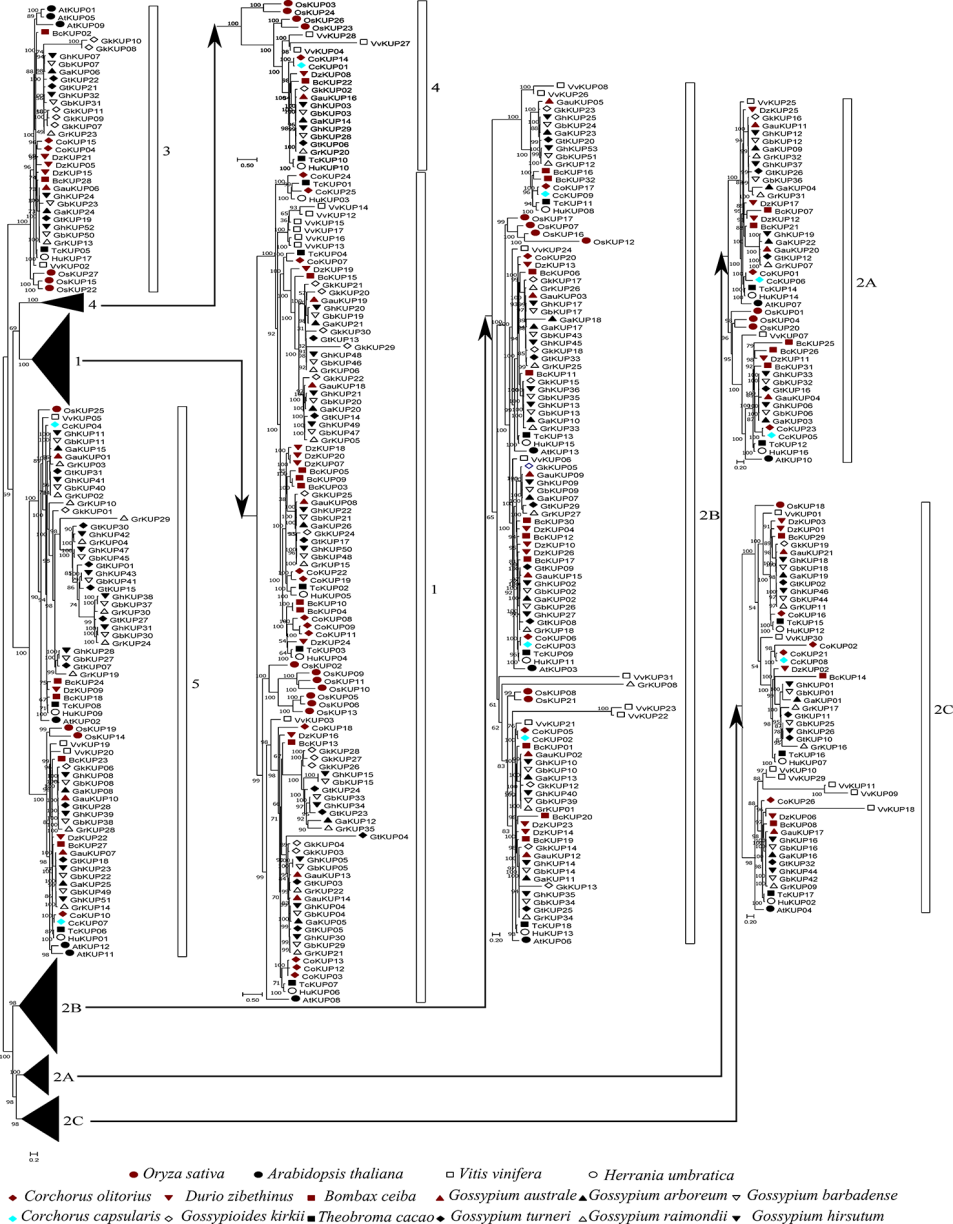
Phylogenetic analysis of the KUP family in 13 Malvaceae species, *Arabidopsis*, grape, and rice. The phylogenetic tree was constructed *via* the ML method. Numbers in the clades represent bootstrap values, and KUP subfamilies are indicated by different letters. KUP members from the investigated species are marked by differently colored shapes.

A total of 448 KUP members were identified in 13 Malvaceae species, *Arabidopsis*, grape, and rice (**Figures 1**, **2**; **Tables S3**, **S4**). The lowest number of KUPs was found in *C. capsularis* (nine members). *Arabidopsis thaliana*, *Herrania umbratica*, and *Theobroma cacao* had less than 20 members. The highest number of members was identified in *G. barbadense* (51) and *G. hirsutum* (53), whereas the diploid donor species *G. arboretum* and *G. raimondii* had 26 and 35 members, respectively. In addition, 24 GbKUPs and 25 GhKUPs were found in the *G. barbadense* At (GbAt) and *G. hirsutum* At (GhAt) subgenomes, respectively. The *G. barbadense* Dt (GbDt) and *G. hirsutum* Dt (GhDt) subgenomes contained 27 GbKUPs and 28 GbKUPs, respectively.

On the basis of previously reported similar classifications (Cheng et al., 2018; Ou et al., 2018; Song et al., 2019), the KUP family can be divided into five subfamilies, which were numbered from 1 to 5. Subfamily 2 could be further classified into three groups, namely, 2A, 2B, and 2C (**Figures 1**, **2**). The KUP members were unevenly distributed throughout each subfamily. In most species, subfamily 2B contained the highest number of KUPs (more than 22%), followed by subfamilies 1 and 5, and subfamily 4 usually had the fewest KUPs (less than 10%). Nearly 70% of the KUP members of *G. barbadense* and *G. hirsutum* belonged to subfamilies 1, 2B, or 5, whereas subfamily 4 of these species contained only two KUPs (**Figure S1**). The number of GbKUPs in subfamilies 1, 3, and 4 was equal to the sum of GaKUPs and GrKUPs. The other subfamilies had low numbers of GbKUPs. GhKUPs also had the same distribution. Most of KUPs that had been lost from *G. barbadense* and *G. hirsutum* were located in the GbDt and GhDt subgenomes. For example, GbKUPs and GhKUPs in subfamily 2B primarily lost three KUPs from the GbDt and GhDt subgenomes.

### Conserved Motifs and Gene Structures of the KUP Family in Cotton

**Figure 3:**
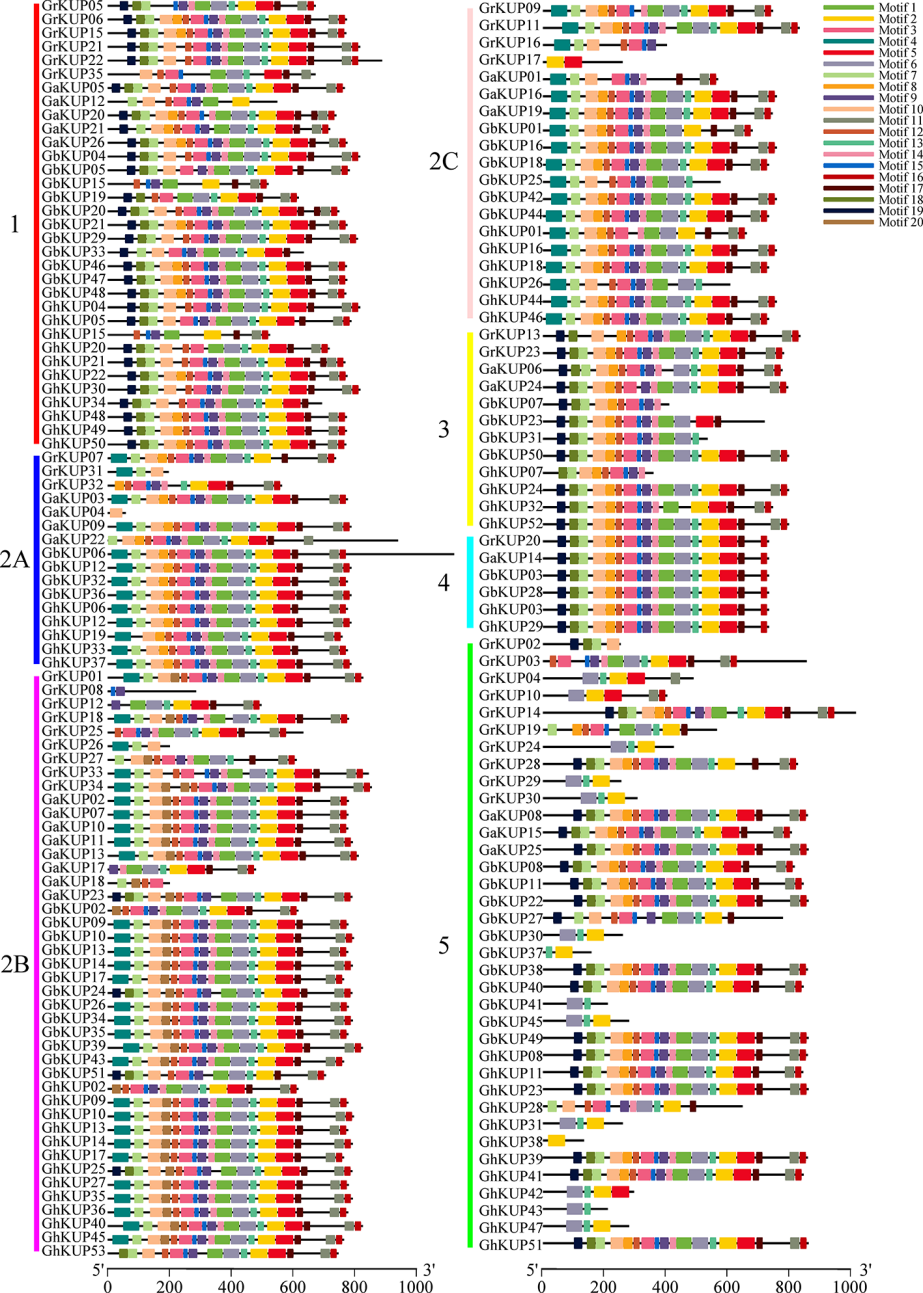
Putative motif distributions of the KUP family in *G. raimondii, G. arboreum, G. barbadense*, and *G. hirsutum*. Different motifs are highlighted by colored boxes. KUP subfamilies are indicated by different colors and letters. Motif location can be estimated by using the scale at the bottom of the figure.

**Figure 4:**
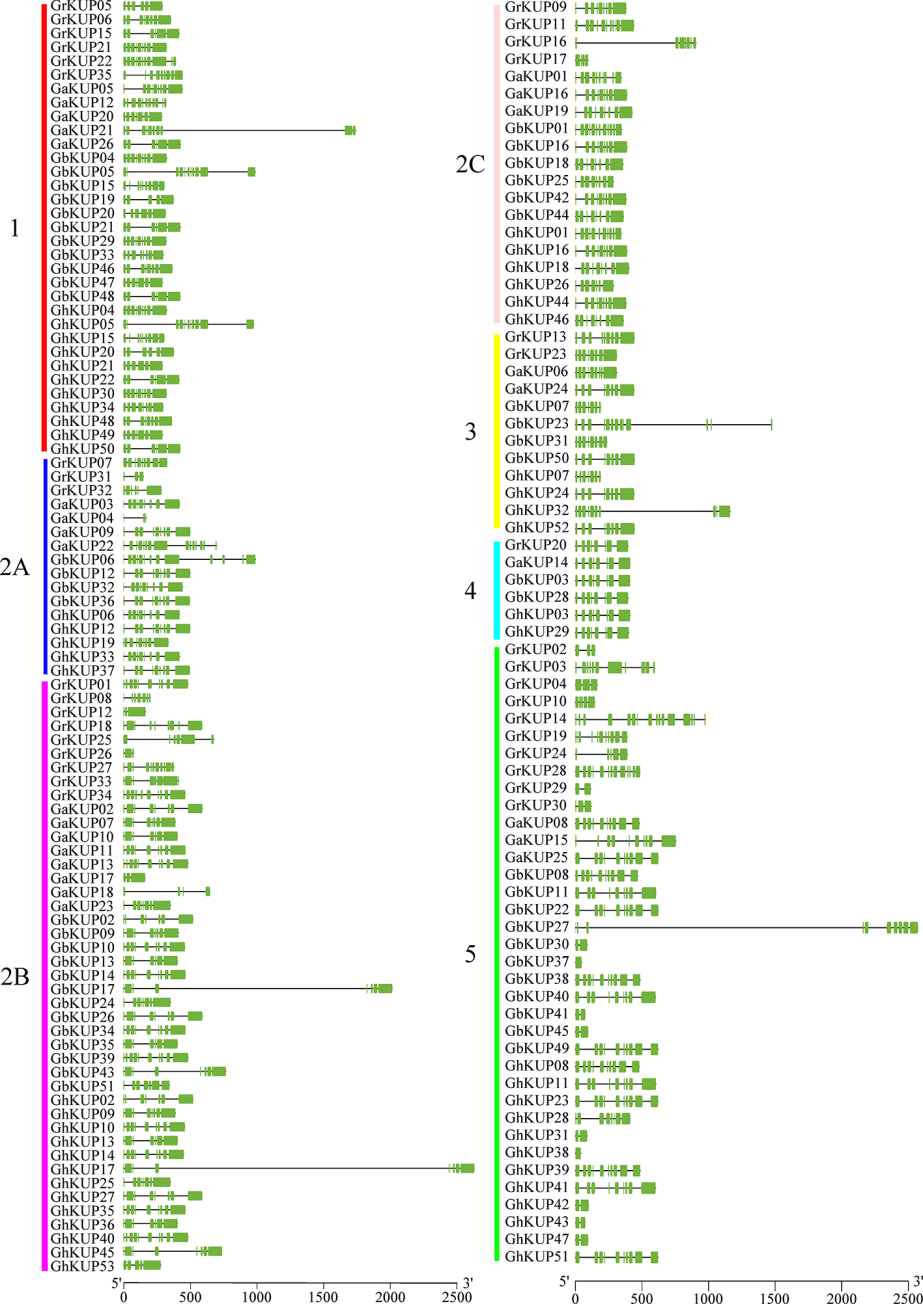
Gene structural analysis of the KUP family in *G. raimondii, G. arboreum, G. barbadense*, and *G. hirsutum*. The green box and the black line represent the exon and intron, respectively. KUP subfamilies are indicated by different colors and letters. The gene structural location can be estimated by using the scale at the bottom of the figure.

All of the KUP members had the conserved KUP domain (**Table S5**). The MEME tool identified 20 putative conserved motifs in GbKUPs, GhKUPs, GaKUPs, and GrKUPs (**Figure 3**; **Table S6**). Consistent with phylogenetic analysis, KUP members could be further classified into five subfamilies, with subfamily 2 containing three groups, on the basis of the distribution of the identified motifs. After CDD search, 18 motifs were annotated as KUP domains. Motifs 8 and 17 lacked any annotations. More than 100 cotton KUP members had 15 motifs. Motifs 4, 8, 18, 19, and 20 existed in some specific subfamilies. Over 75% of the exon numbers of the cotton KUP members ranged from 6 to 10 (**Figure 4**; **Table S7**). The highest exon numbers of GaKUPs, GrKUPs, GbKUPs, and GhKUPs were 16, 15, 13, and 11, respectively. Moreover, the At subgenome of *G. barbadense* and *G. hirsutum* had more highly fragmented KUPs than the Dt subgenome of the two species. The highest exon numbers of the GbAt, GbDt, GhAt, and GhDt subgenomes were 13, 10, 11, and 10, respectively. The exon numbers of the At subgenome were significantly higher than those of the Dt subgenome. Furthermore, gene structure was highly conserved within each subfamily. The KUP members of subfamily 4 had eight exons, and many KUP members with highly fragmented gene structures could be found in subfamily 5.

### Expression Patterns of *GhKUP*s and *GbKUP*s in Different Tissues

**Figure 5:**
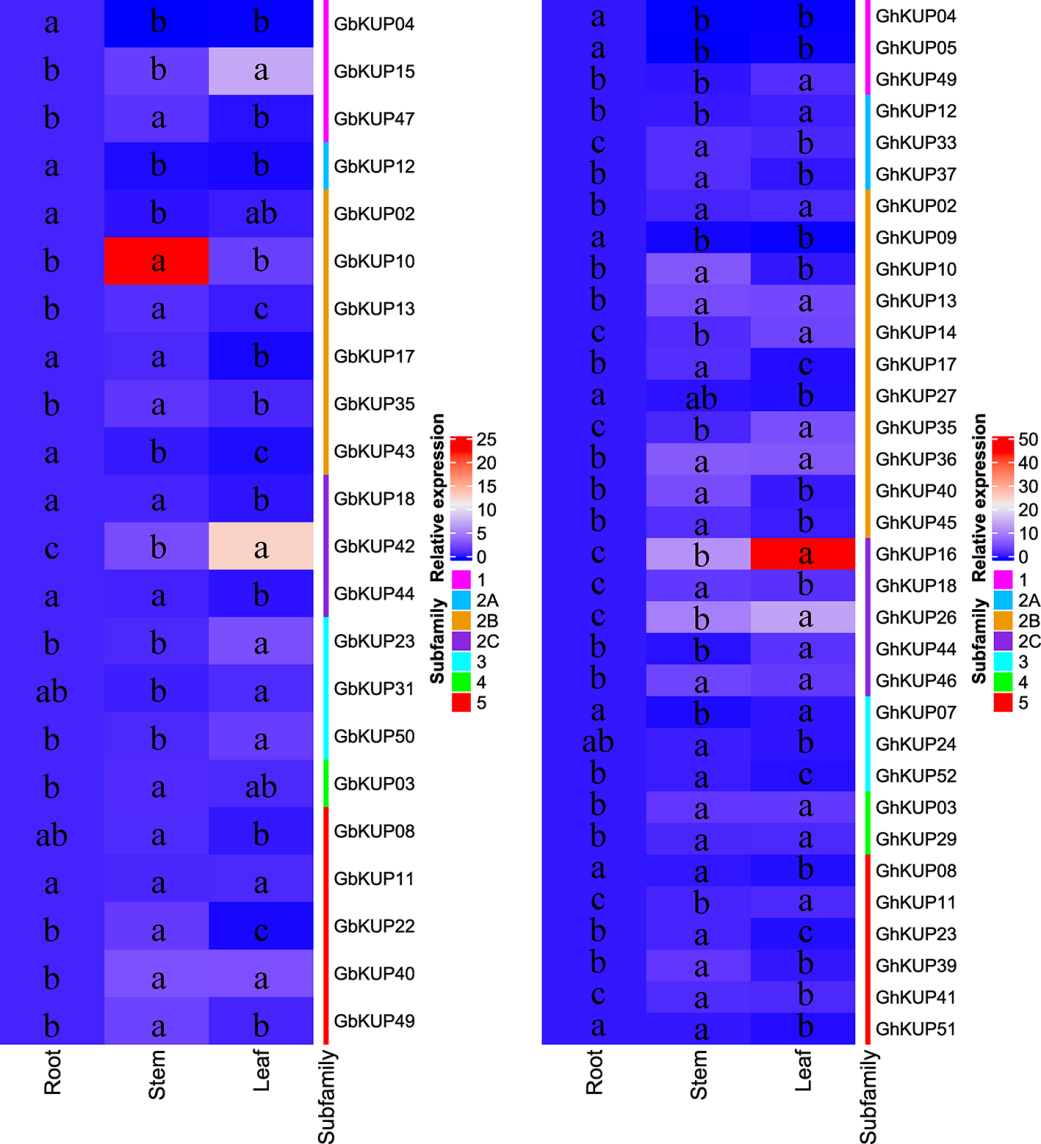
Heat map representation of *GhKUP* and *GbKUP* expression levels in roots, stems, and leaves. Two color bars represent relative expression values and KUP subfamilies. Expression levels with the same letter in each cell do not significantly differ at *P* < 0.05 as revealed by Duncan’s multiple test.

*GhKUPs* and *GbKUPs* showed tissue-specific expression patterns in roots, stems, and leaves (**Figure 5**). In *G. hirsutum*, six *GhKUP*s (*GhKUP02*, *GhKUP03*, *GhKUP13*, *GhKUP29*, *GhKUP36*, and *GhKUP46*) had significantly higher expression levels in stems and leaves than in other tissues. *GhKUP04*, *GhKUP05*, and *GhKUP09* showed the significantly highest expression levels in roots among all the tested tissues. Eleven *GhKUP*s (*GhKUP10*, *GhKUP17*, *GhKUP18*, *GhKUP23*, *GhKUP33*, *GhKUP37*, *GhKUP39*, *GhKUP40*, *GhKUP41*, *GhKUP45*, and *GhKUP52*) exhibited significantly higher expression patterns in stems than in other tissues, whereas GhKUP07 showed opposite expression patterns. Compared with those in other tissues, the expression levels of eight *GhKUP*s (*GhKUP11*, *GhKUP12*, *GhKUP14*, *GhKUP16*, *GhKUP26*, *GhKUP35*, *GhKUP44*, and *GhKUP49*) were significantly higher and those of *GhKUP08*, *GhKUP24*, *GhKUP27*, and *GhKUP51* were significantly lower in leaves. In *G. barbadense*, *GbKUP02*, *GbKUP04*, *GbKUP12*, and *GbKUP43* significantly peaked in roots. *GbKUP15*, *GbKUP23*, *GbKUP31*, *GbKUP42*, and *GbKUP50* showed significantly higher expression patterns in leaves than in other tissues, whereas *GbKUP08*, *GbKUP17*, *GbKUP18*, and *GbKUP44* had the opposite expression patterns. Eight *GbKUP*s (*GbKUP03*, *GbKUP10*, *GbKUP13*, *GbKUP22*, *GbKUP35*, *GbKUP40*, *GbKUP47*, and *GbKUP49*) showed significantly higher expression levels in stems than in other tissues.

### Regulatory Elements of the GhKUP and GbKUP Promoters

**Figure 6:**
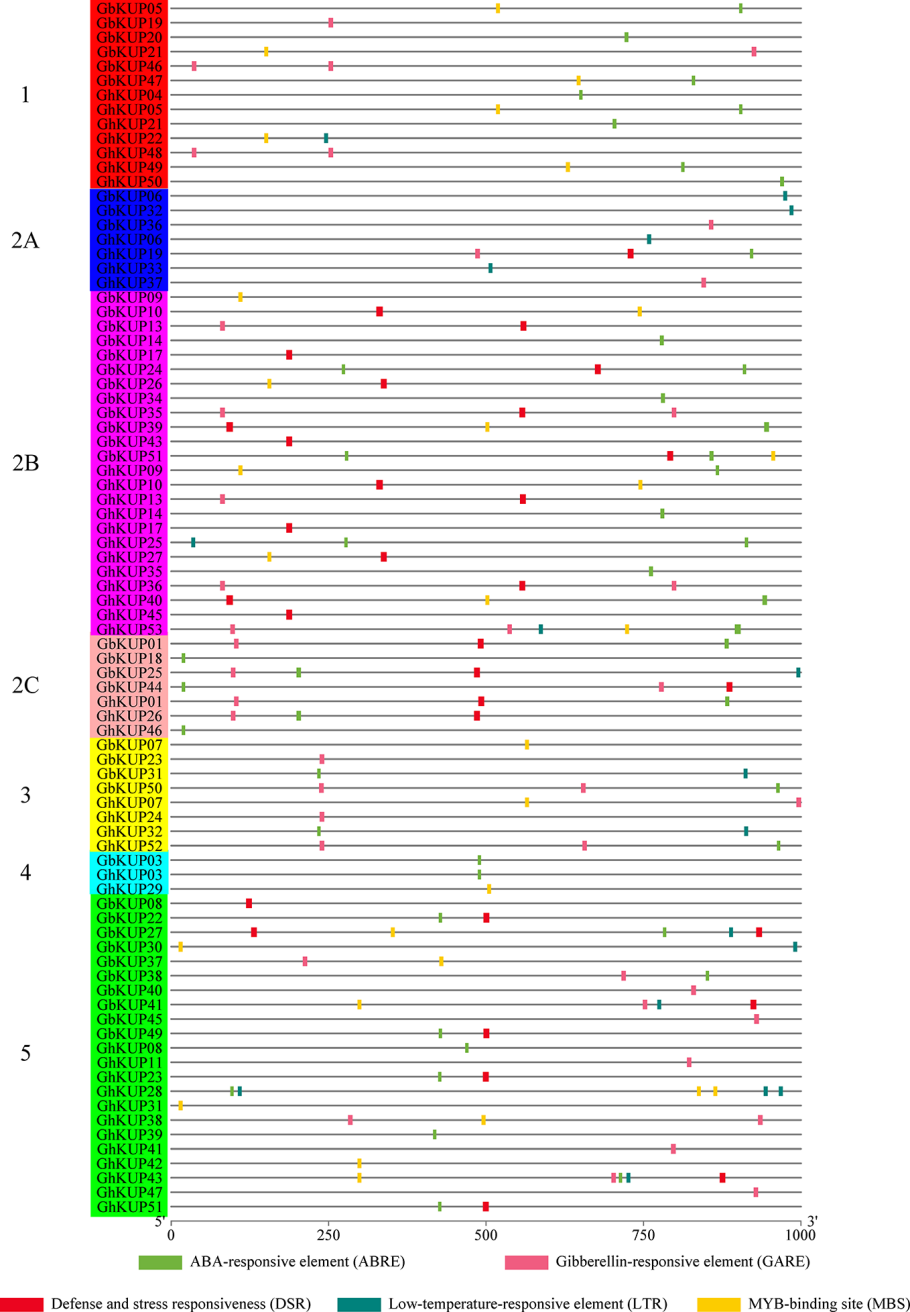
Stress-responsive regulatory elements in the promoter regions of *GhKUP*s and *GbKUP*s. The stress-responsive regulatory elements were predicted in the promoter region of *GhKUP*s and *GbKUP*s by applying the PlantCARE database. The KUP subfamilies are marked by using different colors and letters. The colored boxes represent stress-responsive elements. The location of the stress-responsive elements can be estimated by using the scale at the bottom of the figure.

The regulatory elements of the promoters are crucial. Many stress-responsive regulatory elements, including ABRE, GARE, DSR, LTR, and MBS, were identified in the promoter regions of *GhKUP*s and *GbKUP*s through PlantCARE analysis (**Figure 6**; **Table S8**). Over 80% of the *KUP* promoters in *G. hirsutum* and *G. barbadense* had the above-mentioned elements. The most common element was ABRE (19 *GbKUP*s and 24 *GhKUP*s), followed by GARE (16 *GbKUP*s and 16 *GhKUP*s), DSR (17 *GbKUP*s and 13 *GhKUP*s), and MBS (13 *GbKUP*s and 15 *GhKUP*s). The promoter regions of 44 *KUP*s contained at least two stress-responsive regulatory elements. For example, *GhKUP27* contained DSR and MBS in its promoter region, and ABRE and MBS were found in the promoter region of *GbKUP47*. Moreover, *GbKUP*s and *GhKUP*s from the same subfamily had similar elements in their promoter regions. To illustrate, ABRE, DSR, and MBS were abundant in the promoter regions of *GbKUP*s and *GhKUP*s from subfamily 2B. ABRE, GARE, and MBS existed in most of the promoter regions of *GbKUP*s and *GhKUP*s from subfamily 5.

### Expression Patterns of *GhKUP*s and *GbKUP*s Under Abiotic Stresses

**Figure 7:**
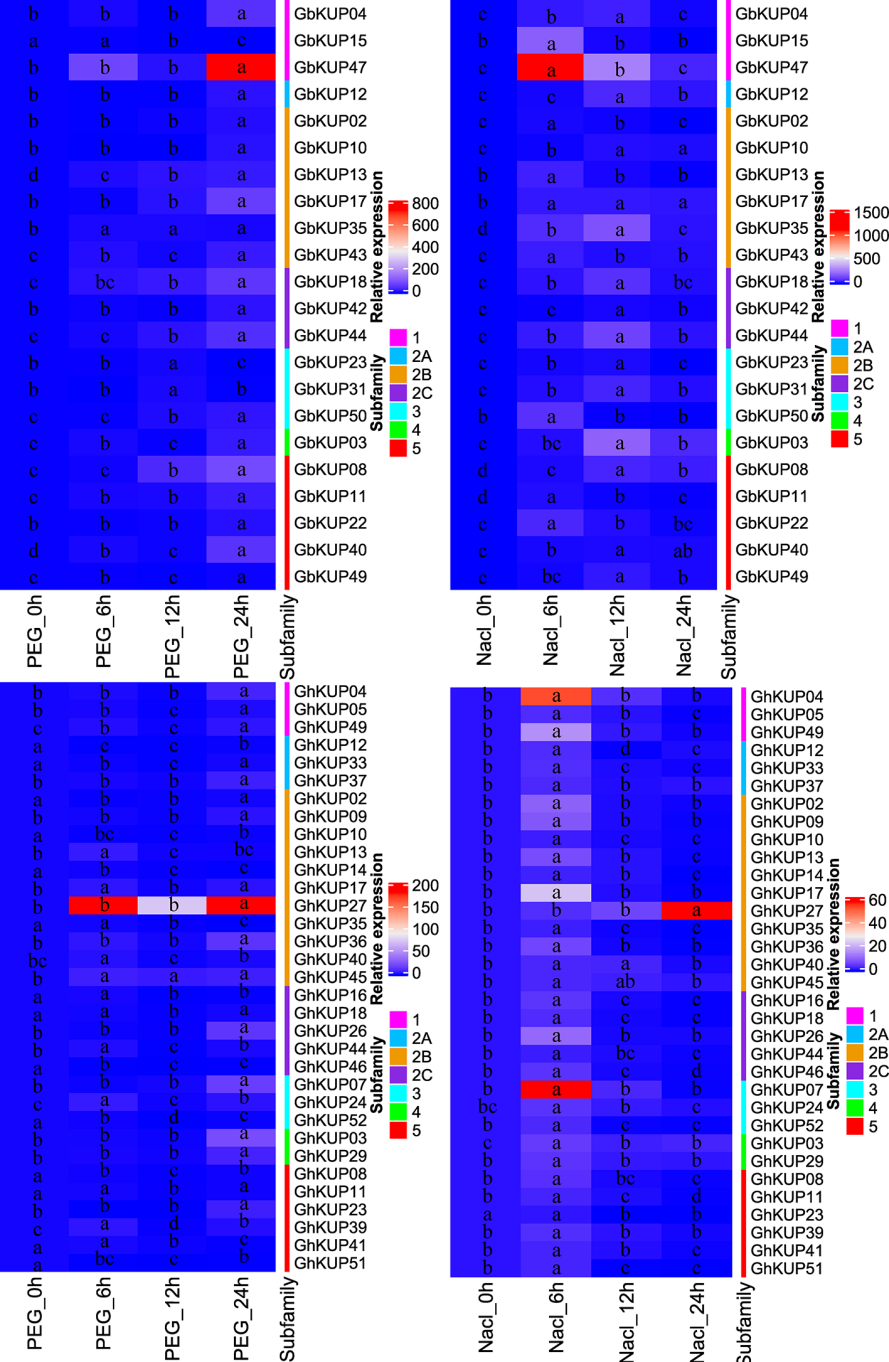
Heat map representation of *GhKUP* and *GbKUP* expression levels under drought and salt stresses. Two color bars represent the relative expression value and KUP subfamily. Expression levels with the same letter in each cell do not significantly differ at *P* < 0.05 as revealed by Duncan’s multiple test.

**Figure 8:**
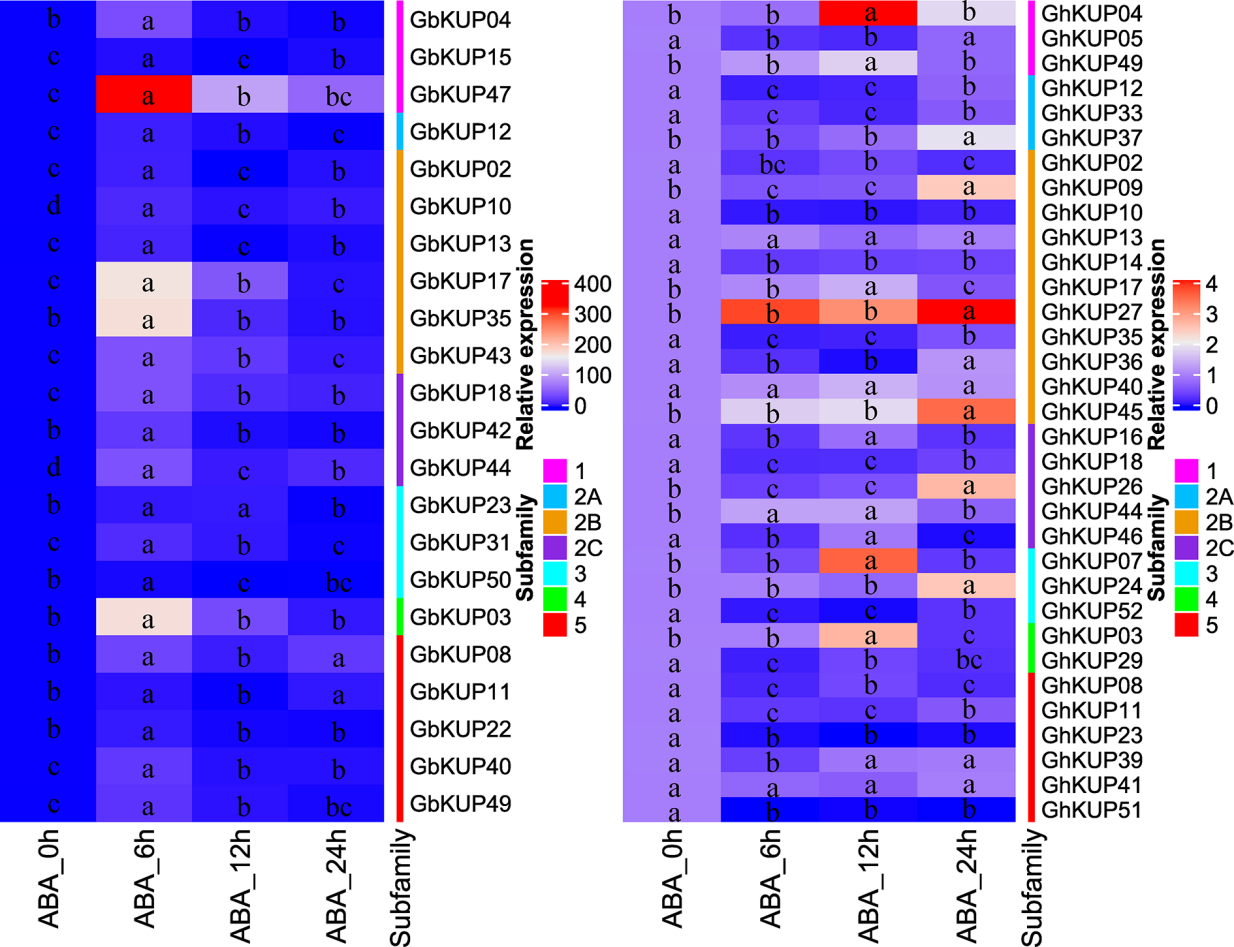
Heat map representation of *GhKUP* and *GbKUP* expression levels under ABA stress. Two color bars represent relative expression values and KUP subfamilies. Expression levels with the same letter in each cell do not significantly differ at *P* < 0.05 as revealed by Duncan’s multiple test.

On the basis of the results for regulatory elements, the expression levels of *GbKUP*s and *GhKUP*s under three abiotic stress conditions (drought, salt, and ABA) were further investigated. Most of the KUPs in *G. hirsutum* and *G. barbadense* exhibited different expression levels after exposure to various stresses (**Figures 7**, **8**). Under drought stress, *GhKUP02*, *GhKUP08*, *GhKUP10*, *GhKUP11*, *GhKUP12*, *GhKUP14*, *GhKUP16*, *GhKUP18*, *GhKUP33*, *GhKUP35*, *GhKUP41*, *GhKUP46*, *GhKUP51*, *GhKUP52*, and *GbKUP15* were significantly downregulated, and others were significantly upregulated (**Figure 7**). *GhKUP13*, *GhKUP17*, *GhKUP24*, *GhKUP39*, *GhKUP40*, *GhKUP44*, *GhKUP45*, *GbKUP23*, *GbKUP31*, and *GbKUP35* were rapidly upregulated after 6 h or 12 h of drought treatment, and others were upregulated after 24 h of drought treatment. In particular, *GhKUP03*, *GhKUP27*, *GbKUP08*, and *GbKUP47* were highly upregulated after 24 h of drought treatment. Under salt stress, all *GbKUP*s and *GhKUP*s showed significantly upregulated expression levels (**Figure 7**). *GhKUP27*, GbKUP10, GbKUP17, and GbKUP40 were upregulated after 24 h of salt treatment, and others were rapidly upregulated after 6 or 12 h of salt treatment. *GhKUP04*, *GhKUP07*, *GhKUP27*, *GbKUP15*, and *GbKUP47* were highly upregulated under salt stress. Under ABA treatment, all *GbKUP*s, particularly *GbKUP03*, *GbKUP17*, *GbKUP35*, and *GbKUP47*, were rapidly upregulated after 6 h, whereas 12 *GhKUP*s (*GhKUP03*, *GhKUP04*, *GhKUP07*, *GhKUP09*, *GhKUP17*, *GhKUP24*, *GhKUP26*, *GhKUP27*, *GhKUP37*, *GhKUP44*, *GhKUP45*, and *GhKUP49*) were significantly upregulated after 12 or 24 h of stress treatment (**Figure 8**). *GhKUP04*, *GhKUP27*, and *GhKUP45* were highly upregulated under ABA treatment. Other *GhKUP*s were significantly downregulated under ABA treatment.H

### Genomic Locations and Gene Duplication of GhKUPs and GbKUPs

*GbKUP*s and *GhKUP*s were located unevenly across chromosomes (**Figures S1**-**S3**; **Table S9**). Chromosomes 6, 7, and 13 in each subgenome lacked *KUP* genes. At05 and Dt04 in the At and Dt subgenomes had the highest number of *KUP*s. Furthermore, *GbKUP*s and *GhKUP*s, except for At11 and Dt5, had the same distribution pattern on each chromosome. In At11 and Dt5, *G. hirsutum* had one more *KUP* member than *G. barbadense*.

**Figure 9:**
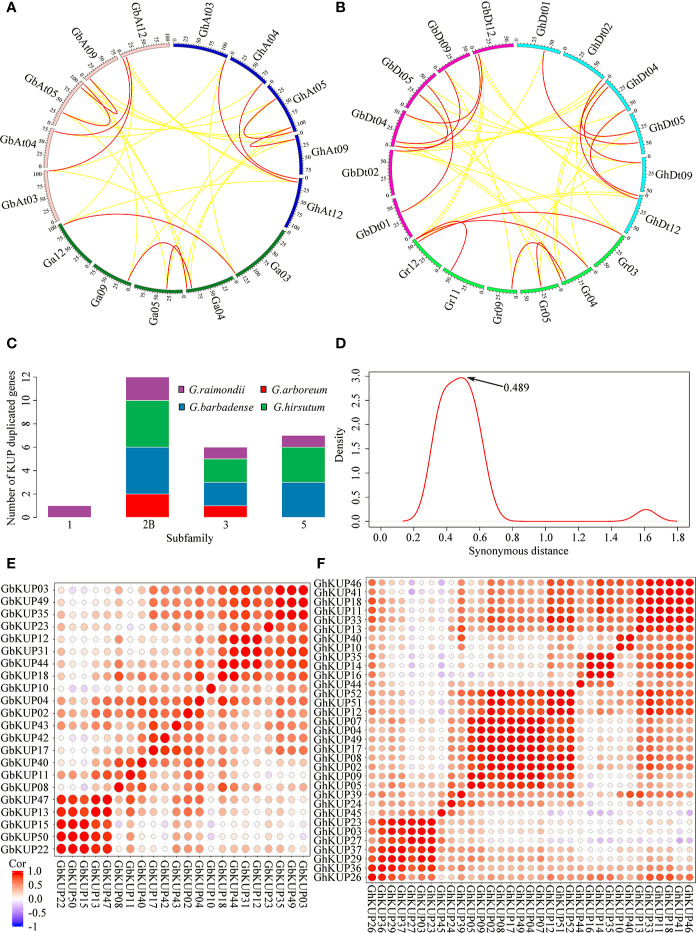
Syntenic analysis, age distribution, and expression relationship of the duplicated *KUP* members in cotton. **(A, B)** Syntenic relationship of the duplicated *GhKUP*s and *GbKUP*s in the A-related genome **(A)** and D-related genome **(B)**. Red lines connect duplicated *KUP*s, and yellow lines connect orthologous genes in other species. The different colored sections of the circles indicate different genomes or subgenomes. **(C)** Subfamily distribution of the duplicated *KUP* members in cotton. **(D)** Age distribution of the duplicated *KUP*s in cotton based on Ks values. The peak value of the duplicated *KUP*s is marked with an arrow. **(E, F)** Heat map of the PCC of the expression profiles of the GbKUP **(E)** and GhKUP **(F)** families.

**Figure 10:**
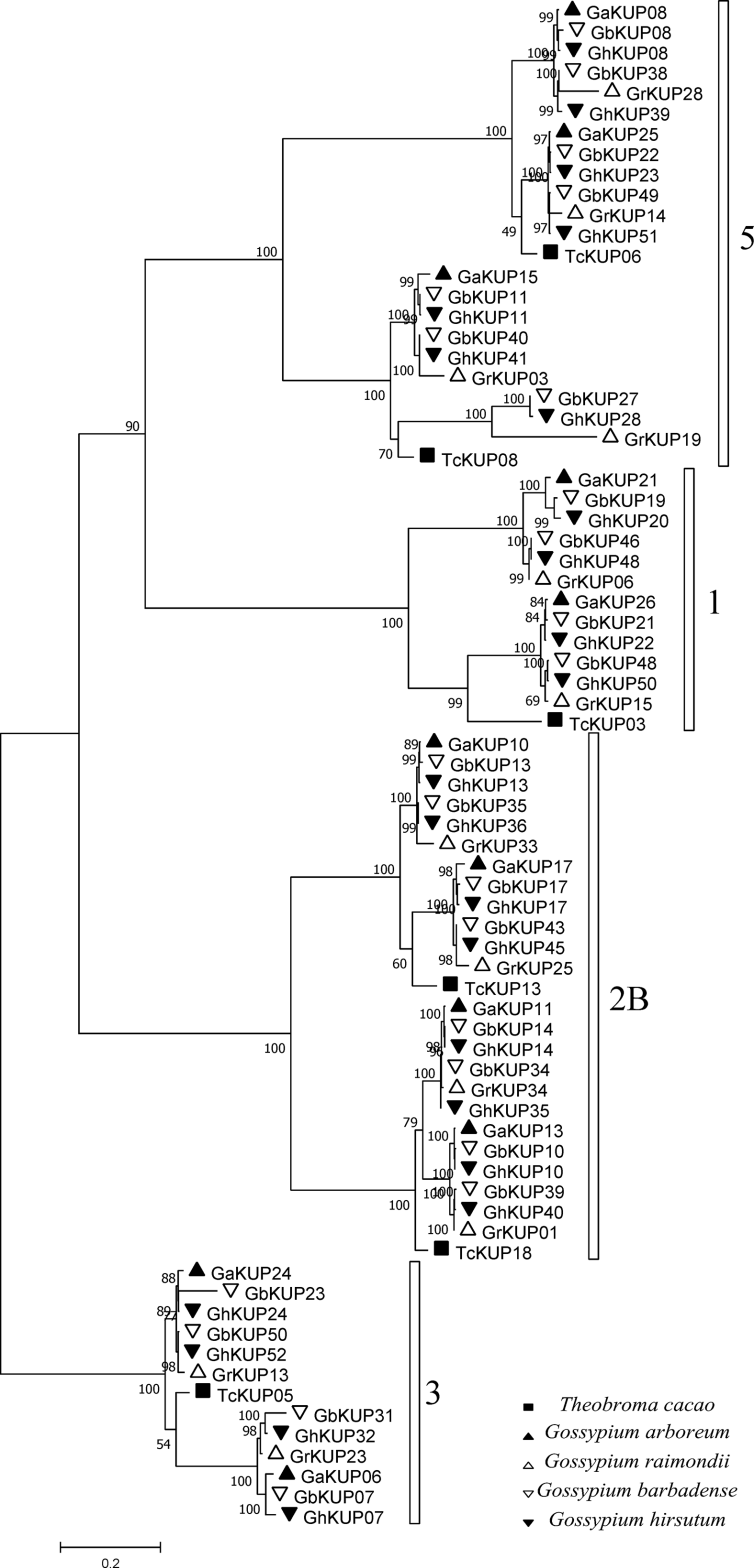
Phylogenetic analysis of duplicated *GaKUP*s, *GrKUP*s, *GhKUP*s, and *GbKUP*s and their orthologous genes in cacao. The phylogenetic tree was constructed *via* the ML method. Numbers in clades represent bootstrap values, and KUP subfamilies are indicated by different letters. KUP members from the investigated species are marked by different shapes.

*G. hirsutum* and *G. barbadense* had each undergone nine KUP family gene duplication events, whereas *G. arboreum* had experienced three and *G. raimondii* underwent five (**Figures 9A, B**; **Table S10**). All duplicated genes were located in different chromosomes, indicating that the gene duplication events were segmental duplication events. The KUP family of cacao, which shares a common ancestor with cotton, underwent one segmental gene duplication event (*TcKUP* 09/13). Duplicated *GhKUP*s and *GbKUP*s belonged to subfamilies 2B, 3, and 5 (**Figure 9C**). Subfamilies 2B and 3 of *G. hirsutum* and *G. barbadense* had twice as many duplicated gene pairs as those of their diploid donor species. However, two additional duplicated gene pairs in subfamily 5 existed only in *G. hirsutum* and *G. barbadense*. Moreover, four and five duplicated gene pairs were distributed in the At and Dt subgenomes, respectively. The orthologous genes of duplicated *GhKUP*s and *GbKUP*s were identified in their corresponding diploid donor species (**Figures 9A, B**; **Table S11**). The orthologous genes of six duplicated *KUP*s (*GaKUP*s and *GrKUP*s) from subfamilies 2B and 3 were duplicated in each allopolyploid species. In subfamily 5, the orthologous genes of two duplicated KUPs (*GhKUP 39/51* and *GbKUP 38*/49) were duplicated in *G. raimondii* (*GrKUP 14/18*), whereas the orthologous genes of four duplicated KUPs (*GhKUP 08/23*, *GhKUP 28/41*, *GbKUP 08*/22, and *GbKUP 27/40*) were not duplicated in *G. arboreum* and *G. raimondii*. The phylogenetic tree of the duplicated *KUP*s in cotton and their orthologous genes in cacao was used to predict the relative time of gene duplication (**Figure 10**). The duplication events of the KUP family were found only in cotton, and orthologous genes in cacao were not duplicated.

The evolutionary distances of nine duplicated *KUP* pairs in *G. hirsutum*, nine duplicated *KUP* pairs in *G. barbadense*, three duplicated *KUP* pairs in *G. arboreum*, and five duplicated *KUP* pairs in *G. raimondii* were calculated (**Figure 9D**; **Table S10**). The Ks values peaked at approximately 0.489, and the divergence time of the duplicated gene pairs corresponded to approximately 94 MYA. Furthermore, the Ka/Ks ratios of all duplicated *KUP* members were less than 1. PCC values were calculated on the basis of the expression levels of *GhKUP*s and *GbKUP*s in different tissues and stresses (**Figures 9E, F**). All of the expression levels of the duplicated gene pairs were positively correlated (PCC > 0), and over 50% of the duplicated gene pairs had highly positively correlated expression levels (PCC > 0.4). For example, the PCC values of the expression levels of the duplicated *GhKUP 13/17* and *GbKUP 35/43* were 0.66 and 0.64, respectively.

### Gene Loss During Cotton KUP Evolution

**Figure 11:**
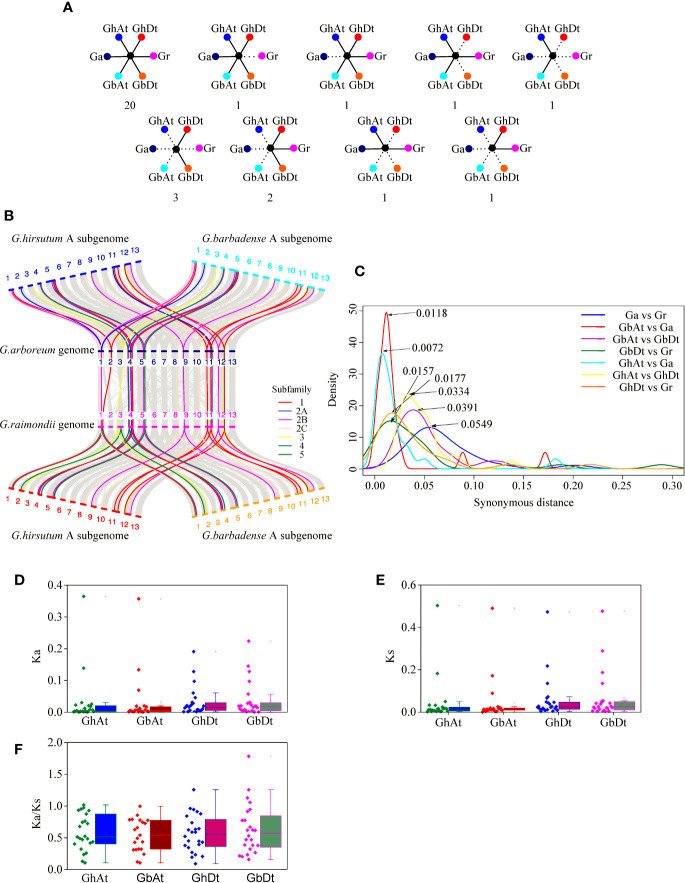
Syntenic and evolutionary analyses of the KUP members of *G. hirsutum* (GhAt and GhDt), *G. barbadense* (GbAt and GbDt), *G. arboretum* (Ga), and *G. raimondii* (Gr). **(A)** Scenarios and numbers of the conserved KUPs in cotton. Solid lines represent extant genes, and dotted lines represent lost genes. The numbers beneath each drawing show the number of gene pairs found in four cotton genomes that fit the corresponding model. From left to right in the first line: KUPs present in all four genomes; KUPs not observed in Gr; KUPs not observed in Ga; KUPs not observed in GhDt and GbDt; and KUPs not observed in GhDt, GbDt, Ga and Gr. From left to right in the second line: KUPs not observed in GhAt, GbAt, Ga, and Gr; KUPs not observed in GhAt, GbAt and Ga; KUPs not observed in GbAt, GbDt, and GhDt; and KUPs not observed in GbAt, GbDt, GhAt, and Ga. **(B)** Microcollinearity analysis of genomic regions from four cotton species. Gray lines link orthologous gene pairs, and orthologous KUP members from different subfamilies are highlighted with different colored lines. **(C)** Ks distribution for the orthologous KUP gene pairs of four cotton species. Peak values for each comparison are marked with arrows. Blue, red, purple, dark green, cyan, yellow, and orange lines represent the Ks distribution of the orthologous KUP gene pairs of Ga and Gr, GbAt and Ga, GbAt and GbDt, GbDt and Gr, GhAt and Ga, GhAt and GhDt, and GhDt and Gr, respectively. **(D–F)** Distribution of Ka values **(D)**, Ks values **(E)**, and Ka/Ks ratio **(F)** between allopolyploid subgenomes and their corresponding ancestral species.

In this study, 31 orthologous gene groups of the KUP family were identified in four cotton species (**Figures 11A, B**; **Table S11**). A total of 20 orthologous gene groups in *G. hirsutum* and *G. barbadense* were also highly conserved in *G. arboreum* and *G. raimondii*, and two orthologous gene groups lost a *GaKUP* or *GrKUP*. In the allopolyploid species, two orthologous genes were found only in the GhAt and GbAt subgenomes, whereas the GhDt and GbDt subgenomes contained five unique orthologous genes. Meanwhile, only two orthologous gene groups were identified in *G. hirsutum* and *G. arboreum* or *G. raimondii*. Furthermore, six and seven *KUP*s were absent from the GhAt and GbAt subgenomes, respectively, whereas three *KUP*s were lost from the GhDt subgenome and four *KUP*s were lost from the GbDt subgenome. A total of eight GaKUPs, five GrKUPs, nine GhKUPs, and 11 GbKUPs were lost from the KUP family. Moreover, the lost *GhKUP*s or *GbKUP*s were distributed unevenly in the KUP subfamily (**Figure 11B**). A total of 13 genes were absent from subfamily 5. Two, three, and two *KUP*s were lost from subfamilies 1, 2A, and 2B, respectively. However, no gene loss occurred in subfamilies 2C, 3, and 4.

### Evolutionary Analysis of the KUP Family in the Cotton Subgenomes

The evolutionary distances (Ka, Ks, and Ka/Ks ratio) of the 159 KUP gene pairs that were orthologous between the allopolyploid species and their diploid donor genomes were calculated (**Table S12**). The Ks values of the KUP members between *G. arboreum* and *G. raimondii* peaked at 0.0549, those between GbAt and GbDt peaked at 0.0391, and those between GhAt and GhDt peaked at 0.0334 (**Figure 11C**). The corresponding divergence times between *G. arboreum* and *G. raimondii* were 10.6 MYA, that between GbAt and GbDt were 7.5 MYA and that between GhAt and GhDt were 6.4 MYA. Ks values of the KUP members between GhAt and GhDt were significantly lower than those between *G. arboreum* and *G. raimondii*. The divergence time between the At subgenome and *G. arboreum* genome was approximately 1.38–2.27 MYA (Ks peaked at 0.0072 between the GhAt subgenome and *G. arboreum* genome and 0.0118 between the GbAt subgenome and *G. arboreum* genome). The divergence time between the Dt subgenome and *G. raimondii* genome was predicted to be 3.02–3.40 MYA (Ks peaked at 0.0157 between the GbDt subgenome and *G. raimondii* genome and 0.0177 between the GhDt subgenome and *G. raimondii* genome). In addition, the Ka, Ks, and Ka/Ks ratios between the At or Dt subgenome and their corresponding diploid donor genomes were similar in *G. hirsutum* and *G. barbadense* (**Figures 11D–F**).

## Discussion

In this study, we identified 51 GbKUPs and 53 GhKUPs in *G. hirsutum* and *G. barbadense*, respectively. These numbers were higher than those reported for any other investigated species (**Figure 1**; **Tables S3**, **S4**). The increase in the KUP members of *G. hirsutum* and *G. barbadense* was associated with the allopolyploidization of the A and D diploid donor species (Wendel, 1989; Paterson et al., 2012; Wang et al., 2017). Although the A-related genome was larger than the D-related genome, the number of KUP members in the A-related genome was lower than that in the D-related genome likely because of the presence of additional LTR-type retrotransposons in the A-related genome (Li et al., 2014; Li et al., 2015; Du et al., 2018). Additional LTR-type retrotransposons in the A-related genome might have resulted in the reduced protein-coding capacities of the A-related genome. Thus, the number of KUP members in the A-related genome was not larger than that in the D-related genome. The low distribution of members in the A-related genome is also exhibited by cotton CDK and PP2C families (Magwanga et al., 2018; Shazadee et al., 2019). Moreover, in contrast to the classification provided by previous studies, we found evidence for the existence of five KUP subfamilies with three groups in subfamily 2 (**Figures 1**, **2**). The subfamily that was regarded as group 3B in previous reports was identified as subfamily 5 in the present study due to its independent phylogenetic relationship with subfamily 3 (Cheng et al., 2018; Ou et al., 2018; Song et al., 2019). Meanwhile, each subfamily of *G. hirsutum* and *G. barbadense* had similar numbers of KUP members. Subfamilies 1, 2B, and 5 had the highest number of members, whereas subfamily 4 contained the lowest number of KUPs. Similar distribution patterns of KUP members have been observed in other species (Cheng et al., 2018; Ou et al., 2018; Song et al., 2019). The distribution of conserved motifs, gene structures, and regulatory elements within subfamilies largely supported the results of phylogenetic analysis (**Figures 3**, **4**, **6**). Moreover, *GbKUP*s and *GhKUP*s were unevenly distributed across cotton chromosomes, with the largest number of *KUP*s in At05 and Dt04 (**Figures S1**-**S3**; **Table S9**). Uneven chromosomal distributions have been also reported for the NAC, PP2C, and CDK gene families in cotton (Fan et al., 2018; Magwanga et al., 2018; Shazadee et al., 2019).

Expression patterns can reflect the possible functions of *GhKUP*s and *GbKUP*s. The high expression levels of most *GhKUP*s and *GbKUP*s in leaves and stems were suggestive of their roles in K^+^ transport (**Figure 5**). The expression levels of the KUP family in cotton were similar to those in other species (Ahn et al., 2004; Gupta et al., 2008; Ou et al., 2018). Three *GhKUP*s and four *GbKUP*s showed significantly high expression levels only in roots. These genes may participate in K+ absorption. An orthologous gene of *GhKUP04*, *GhKUP05*, and *GbKUP04* in rice (*OsKUP02*) plays a major role in K+ absorption by roots (Yang et al., 2014), and another orthologous gene of *GbKUP12* in *Arabidopsis* (*AtKUP10*) may be related to the absorption of K+ and the establishment of root tip growth (Rigas et al., 2001). Moreover, the promoter regions of *GhKUP*s and *GbKUP*s contained numerous stress-responsive regulatory elements (**Figure 6**; **Table S8**). The significantly different expression levels of *GbKUP*s and *GhKUP*s under drought, salt, and ABA stresses indicated their possible functions in abiotic stress response (**Figures 7**, **8**). Many KUP members in *Arabidopsis* (e.g., *AtKUP03*, *AtKUP06*, *AtKUP08*, and *AtKUP13*) and rice (e.g., *OsKUP02*, *OsKUP04*, *OsKUP10*, *OsKUP11*, *OsKUP13*, and *OsKUP17*) can participate in abiotic stress response (Ahn et al., 2004; Osakabe et al., 2013; Yang et al., 2014; Chen et al., 2015; Shen et al., 2015). These results indicated that the KUP family might be related to K+ uptake and efflux through ABA regulation during osmotic adjustment (Very and Sentenac, 2003; Fujita et al., 2011). In addition, *GhKUP27* and *GbKUP47* were highly upregulated under drought, salt, and ABA treatments. Therefore, *GhKUP27* and *GbKUP47* might play a vital role in abiotic stresses. Their corresponding orthologous genes in *Arabidopsis* and rice can respond to multiple abiotic stresses (Osakabe et al., 2013; Yang et al., 2014; Shen et al., 2015).

The expansion of the KUP family in *G. hirsutum* and *G. barbadense* was analyzed **(****Figures 9A, B**; **Table S10**). Duplication events occurred more frequently in cotton than in cacao. The greater number of duplication events in cotton than in cacao partially explained the increase in the KUPs of four cotton species (**Figure 1**). Meanwhile, the KUP members of the two subgenomes expanded asymmetrically. Compared with the At subgenome, the Dt subgenome had more duplicated gene pairs, which resulted in additional *KUP* members. Furthermore, duplication events were not random across the KUP family, and expansion was preferred in subfamilies 2B, 3, and 5 (**Figures 9C**, **10**). The orthologous genes of *GhKUP*s and *GbKUP*s that were duplicated in subfamilies 2B and 3 were also duplicated in their diploid donor species (**Figures 9A, B**; **Table S11**). In subfamily 5, the four orthologous genes of duplicated *GhKUP*s and *GbKUP*s were absent from the syntenic blocks of the diploid donor species. These results may be associated with genomic fractionation during cotton evolution (Wang et al., 2016). Preferential subfamily expansion was found in other families (Fan et al., 2018; Liu et al., 2019; Shazadee et al., 2019). Moreover, segmental duplication dominated the expansion of *KUP* genes in cotton. In this study, the Ks peak was approximately 0.489, suggesting that the divergence times of all of the duplicated *KUP*s, except *GrKUP06/15*, corresponded to a paleohexaploidization event (**Figure 9D**; **Table S10**). Most of the *KUP*s that were duplicated in the allopolyploid species could be used to identify their orthologous duplicated genes in their diploid donor species (**Figures 9A, B**; **Table S11**). However, their orthologous genes were not duplicated in cacao. Phylogenetic analysis indicated that the duplication events of the KUP family might have occurred in the cotton ancestor (**Figure 10**). Thus, the duplicated *KUP*s in *G. hirsutum* and *G. barbadense* have happened in a common ancestor of *Gossypium*, and all of the duplication events occurred during the cotton paleohexaploidization event. In addition, the Ka/Ks ratios were less than 1 for all duplication events, indicating that the retained KUP members mainly experienced purifying selection (**Table S10**). The positive correlation among the expression levels of the duplicated *GhKUP*s and *GbKUP*s suggested that the duplicated genes maintained their original or similar functions during sequential evolution (**Figures 9E, F**) (Adams et al., 2003). Thus, subfunctionalization was the evolutionary fate of the duplicated *KUP*s in the cotton genome. The NAC and HMGS families underwent a similar evolutionary fate (Fan et al., 2018; Liu et al., 2019).

Postpolyploidization genomes may be reshuffled extensively partly due to an increase in homologous chromosomes (Gordon et al., 2009; Wang et al., 2009). Genome restructuring leads to large-scale chromosome loss, fusion, and fission (Buggs et al., 2012). After allopolyploid formation, *G. hirsutum* and *G. barbadense* tended to regain diploid heredity (Wang et al., 2016). More KUP members were lost from *G. hirsutum* and *G. barbadense* than from the diploid species (**Figure 11A**; **Table S11**). Gene loss accounted for the low numbers of the KUP members of *G. barbadense*. At11 and Dt5 in *G. barbadense* lost one KUP more than those in *G. hirsutum*. Meanwhile, gene loss was asymmetric in the At and Dt subgenomes. The high rate of gene losses in the At subgenome might have resulted from intensive human selection after domestication (Zhang et al., 2015; Hu et al., 2019). The high retention rates in the Dt subgenome might be related to abiotic stress tolerance (Zhang et al., 2015). The expression profile results also suggested that *GhKUP*s and *GbKUP*s could respond to abiotic stresses (**Figures 7**, **8**). Furthermore, different KUP subfamilies had different loss rates (**Figure 11B**). Subfamily 5 showed the highest gene loss rate, and subfamilies 2C, 3, and 4 did not lose any genes. The different loss rates shown by KUP families resulted in unequal subfamily distributions.

The *Gossypium* genus underwent two major events: diploid species divergence (5–10 MYA) and interspecific hybridization (1–2 MYA; Zhang et al., 2015; Hu et al., 2019). The Ks distribution values provided evidence for the occurrence of these two major events in the KUP family (**Figure 11C**; **Table S12**). The Ks values between the GhAt and GhDt subgenomes were significantly lower than those between the *G. arboreum* and *G. raimondii* genomes. The significantly reduced Ks values might result from widespread homologous chromosome recombination and crop domestication in *G. hirsutum* (Wang M. et al., 2019). Moreover, the divergence time between the At subgenomes and *G. arboretum* genome was similar to that between the Dt subgenomes and *G. raimondii* genome (**Figures 11D–F**). The At and Dt subgenomes contributed to fiber improvement and stress tolerance traits, respectively (Zhang et al., 2015). Thus, the KUP members of *G. hirsutum* and *G. barbadense* evolved fiber-improvement and stress-tolerant physiologies or phenotypes after domestication at the same time.

In summary, 51 GbKUPs and 53 GhKUPs were identified in *G. hirsutum* and *G. barbadense*, respectively. The KUP family could be divided into five subfamilies with subfamily preference. The duplication events of the KUP family in the cultured allopolyploid species originated from their diploid donor species and occurred during the cotton paleohexaploidization event. Subfunctionalization was the evolutionary fate of duplicated *GhKUP*s and *GbKUP*s. The KUP members in *G. hirsutum* and *G. barbadense* showed tissue-specific expression patterns and could respond to various stresses. Moreover, the KUP family in the At and Dt subgenomes of the allopolyploid species underwent asymmetric evolution. Meanwhile, *G. hirsutum* and *G. barbadense* exhibited conserved and divergent KUP evolution. The present study provided a comprehensive understanding of the KUP family in allopolyploid cotton species.

## Data Availability Statement

The datasets presented in this study can be found in online repositories. The names of the repository/repositories and accession number(s) can be found in the article/**Supplementary Material**.

## Author Contributions

KF, JH, and WXL designed the research. KF, ZM, JZ, YC, and ZL performed the experiments. KF, ZM, WWL, and YZ analyzed the data. KF, JH, and WXL wrote the paper with contributions from all the authors.

## Funding

This work was supported by the National Natural Science Foundation of China (31471567; 31671763; 31701470), China Postdoctoral Science Foundation (2017M610388; 2018T110637), China Scholarship Council (Grant 201708350002 to KF), Fujian Provincial Natural Science Foundation of China (2017J01439), Education Department of Fujian Province of China (JZ160436), Outstanding Youth Scientific Fund of Fujian Agriculture and Forestry University (xjq201917), and Fujian-Taiwan Joint Innovative Centre for Germplasm Resources and Cultivation of Crop (2015-75. FJ 2011 Program, China).

## Conflict of Interest

The authors declare that the research was conducted in the absence of any commercial or financial relationships that could be construed as a potential conflict of interest.
