# Supplementary material for: Molecular Evolution and Expansion of the KUP Family in the Allopolyploid Cotton Species Gossypium hirsutum and Gossypium barbadense
Source: Front Plant Sci. 2020 Sep 30;11:545042. doi: 10.3389/fpls.2020.545042 (PMC7554350; doi:10.3389/fpls.2020.545042)
Supplement: Supplementary file 1 [file DataSheet_1.pdf]

Table S1 Retrieval of the sequences of plant species used in this study.

Family	Species	Data source
Brassicaceae	*Arabidopsis thaliana*	http://www.arabidopsis.org/
Poaceae	*Oryza sativa*	http://www.phytozome.net/
Vitaceae	*Vitis vinifera*	http://www.phytozome.net/
Malvaceae	*Bombax ceiba*	http://magen.whu.edu.cn
Malvaceae	*Corchorus capsularis*	http://magen.whu.edu.cn
Malvaceae	*Corchorus olitorius*	http://magen.whu.edu.cn
Malvaceae	*Durio zibethinus*	http://magen.whu.edu.cn
Malvaceae	*Gossypium arboreum*	https://www.cottongen.org/
Malvaceae	*Gossypium australe*	https://www.cottongen.org/
Malvaceae	*Gossypium barbadense*	https://www.cottongen.org/
Malvaceae	*Gossypium hirsutum*	https://www.cottongen.org/
Malvaceae	*Gossypioides kirkii*	https://www.cottongen.org/
Malvaceae	*Gossypium raimondii*	https://www.cottongen.org/
Malvaceae	*Gossypium turneri*	https://www.cottongen.org/
Malvaceae	*Herrania umbratica*	http://magen.whu.edu.cn
Malvaceae	*Theobroma cacao*	http://www.phytozome.net/

Table S2 PCR primers used in this study.

Gene name	Forward primer (5'-3')	Reverse primer (5'-3')
EF1α	AGACCACCAAGTACTACTGCAC	CCACCAATCTTGTACACATCC
GbKUP02	GGGTTCCTATTGCCCTTGCT	CGAGCTCAGTGTGGACAAGT
GbKUP03	TGGATTTACAGTCAGGGGCG	CGGTGGTATTGTGGAGAGGG
GbKUP04	AGCAGGTATGGCCGAGAATG	ATGAAAGTCTGCCAGCCTCC
GbKUP08	ACATGCCTTTCAGACCCTCG	CCTTAGCATGCCGGCAAATC
GbKUP10	ACGGTAGAGAGTGTGACGGA	CGCGGCAATTCCTTCTCAAG
GbKUP11	GAGTCAAGGTGGGTGGATGG	TGACCATGAGCACCAGCAAT
GbKUP12	CGGTGAAGGTGGCACTTTTG	GAAGGGCTGTCCTTAACGCT
GbKUP13	GCTTGGGTTCCTATTGCCCT	GGATCCCGGAAACAAGCTCA
GbKUP15	CCTGCACCAATTCAGGGAGT	CCATCATTTGTGCCTCCCCT
GbKUP17	GGTTCCCATAGCCCTTGCTT	CGAACGATACCCAGAGTCGG
GbKUP18	TTGCAACGATGGTTGGGAGT	ACACGTGGGAAGCACTTCAA
GbKUP22	AAGCGATGGCGATGAAGACA	AACCATGCTCCGCATCAGAA
GbKUP23	TCGCTCTATCCCTGAGAGCA	CCACAGCTGTCCCATAAGCA
GbKUP31	GCCCTTGCACATGGTTGTTT	ACCACTGCTGTCCCATAAGC
GbKUP35	TCCCGGAGATCAACTGGACT	CTGATGCATTTCCCATGCGT
GbKUP40	GAGGGGTGAACGAGTCAAGG	TGTCACCATACACAACCCCG
GbKUP42	TGCTCCGATTGTCACAGCAT	TGCAAGAACCAAGCAGGGAT
GbKUP43	GGTTCCCATAGCCCTTGCTT	GATCCCGGAAACTAGCTCGG
GbKUP44	GCGTGCAAAGTTGGGTCTTT	CCCCAGCAGCACAAGTAGAA

GbKUP47	ACAGAAGAGCCAATTCGCCA	CGTCTGTGAGGGCAGATGTT
GbKUP49	ATGTGCAACAGGGTCTGAGG	GGCTGCTCTTCACCACTAGG
GbKUP50	AAACACAGTGGTGAGGGCAA	ACCTGCCCTACGTTCAACAG
GhKUP02	GGGTTCCTATTGCCCTTGCT	CGAGCTCAGTGTGGACAAGT
GhKUP03	ATGGGTGCATGGACCTTGAG	TACAGAGGATGGTACCGCCA
GhKUP04	AGCAGGTATGGCCGAGAATG	ATGAAAGTCTGCCAGCCTCC
GhKUP05	AACAAAGCAGGTATGGCCGA	GCCCGCGATAACTTTCGTTC
GhKUP07	CCTCACACTTATCCCGCTCC	AGCTCCTCATCGGTCGTTTG
GhKUP08	ACATGCCTTTCAGACCCTCG	TGACCTTAGCATGCTGGCAA
GhKUP09	AGAGTTGCGGGAACTTACGG	CGTCGAGACATGAGGTACGG
GhKUP10	ACGGTAGAGAGTGTGACGGA	CGCGGCAATTCCTTCTCAAG
GhKUP11	GAGTCAAGGTGGGTGGATGG	TGACCATGAGCACCAGCAAT
GhKUP12	CGGTGAAGGTGGCACTTTTG	GAAGGGCTGTCCTTAACGCT
GhKUP13	GCTTGGGTTCCTATTGCCCT	GGATCCCGGAAACAAGCTCA
GhKUP14	TCCTGCCTTCCATTGTGTCC	ATAAGACCGATGAGCAGCCG
GhKUP16	CCCTGCCTGGTTCTTGCATA	CAGCAGCAAAAGTTGCCACA
GhKUP17	GGTTCCCATAGCCCTTGCTT	CGAACGATACCCAGAGTCGG
GhKUP18	TTGCAACGATGGTTGGGAGT	ACACGTGGGAAGCACTTCAA
GhKUP23	AAGCGATGGCGATGAAGACA	AACCATGCTCCGCATCAGAA
GhKUP24	CAATGCATGGGAGCATGACG	CCCAGAATGTGTACCACCCC
GhKUP26	TTGCACATCTTGTGGCAAGC	AAACGGAATTCGGGAGGACC
GhKUP27	CTTGTCCACACTGCTCTCGT	GCCTGTACTCCCTAGGTCCA

GhKUP29	AGTATGGTTGCCAGAAGGGC	GGGAACGGTGCTCTTAGTCC
GhKUP33	TGGCAACTTTGGCTGCAATC	AAACCCAACTGTGACTGCCA
GhKUP35	GCTCCCTATTCTCTTGGCCC	CGATGGAAGGCAGGAAGGTT
GhKUP36	TCCCGGAGATCAACTGGACT	AGCAAGGGCAATAGGAACCC
GhKUP37	AACCTGCCAGCGTTCCATAA	AGCTGCTCCTCAAAATCCCC
GhKUP39	GAGCTAAACCCCAACTCCCC	GCAATACCCCTCCTGCAGTT
GhKUP40	TCTTGGAAGACGGCATTGCT	TCCGCTCGAAGAACCACAAA
GhKUP41	GAGGGGTGAACGAGTCAAGG	TGTCACCATACACAACCCCG
GhKUP44	TGCTCCGATTGTCACAGCAT	TGCAAGAACCAAGCAGGGAT
GhKUP45	GGTTCCCATAGCCCTTGCTT	GATCCCGGAAACTAGCTCGG
GhKUP46	GCCCACCAGAGTTCTGTCTC	ACGGACTTGATTGCTGCTGA
GhKUP49	ACAGAAGAGCCAATTCGCCA	CGTCTGTGAGGGCAGATGTT
GhKUP51	ATGTGCAACAGGGTCTGAGG	GGCTGCTCTTCACCACTAGG
GhKUP52	AAACACAGTGGTGAGGGCAA	ACCTGCCCTACGTTCAACAG

Table S3 KUP proteins identified in *G. raimondii*, *G. arboreum*, *G. barbadense*, and *G. hirsutum*.

Name	Locus name	Subfamily	E-value	Score	Bias
GrKUP01	D5_v1_pred_00003002	2B	6.00E-193	647.4	19.6
GrKUP02	D5_v1_pred_00003600	5	3.50E-37	133.3	0.2
GrKUP03	D5_v1_pred_00003601	5	6.10E-115	390	21.8
GrKUP04	D5_v1_pred_00003691	5	1.10E-36	131.7	0.9
GrKUP05	D5_v1_pred_00005846	1	2.00E-141	477.4	12.1
GrKUP06	D5_v1_pred_00007564	1	8.70E-193	646.9	28.9
GrKUP07	D5_v1_pred_00008113	2A	1.40E-167	563.7	23.4
GrKUP08	D5_v1_pred_00009562	2B	5.20E-15	60.2	0.5
GrKUP09	D5_v1_pred_00011392	2C	1.80E-164	553.5	26.3
GrKUP10	D5_v1_pred_00014313	5	8.10E-08	36.4	1.1
GrKUP11	D5_v1_pred_00014856	2C	4.60E-177	595	19.4
GrKUP12	D5_v1_pred_00015425	2B	2.30E-81	279.2	9.1
GrKUP13	D5_v1_pred_00018102	3	6.60E-175	587.9	25.5
GrKUP14	D5_v1_pred_00018453	5	1.10E-146	494.7	22
GrKUP15	D5_v1_pred_00018504	1	2.10E-197	662.1	22.3
GrKUP16	D5_v1_pred_00022827	2C	1.70E-82	282.9	7.5
GrKUP17	D5_v1_pred_00022828	2C	2.20E-11	48.2	0
GrKUP18	D5_v1_pred_00023479	2B	6.90E-152	511.9	14.2
GrKUP19	D5_v1_pred_00023487	5	5.60E-91	310.9	25.4
GrKUP20	D5_v1_pred_00023562	4	5.10E-188	631.1	21.8

GrKUP21	D5_v1_pred_00024076	1	3.70E-190	638.2	19.5
GrKUP22	D5_v1_pred_00024080	1	3.50E-189	635	20.1
GrKUP23	D5_v1_pred_00024651	3	2.60E-185	622.2	24.3
GrKUP24	D5_v1_pred_00026405	5	3.80E-25	93.6	5.2
GrKUP25	D5_v1_pred_00028082	2B	2.80E-135	457.1	19.9
GrKUP26	D5_v1_pred_00028083	2B	1.30E-43	154.6	0.1
GrKUP27	D5_v1_pred_00036343	2B	1.80E-126	428	16.8
GrKUP28	D5_v1_pred_00036687	5	5.70E-145	489.1	14.1
GrKUP29	D5_v1_pred_00037449	5	2.60E-19	74.4	6.6
GrKUP30	D5_v1_pred_00037468	5	1.80E-24	91.4	6.4
GrKUP31	D5_v1_pred_00037809	2A	8.90E-39	138.6	0.2
GrKUP32	D5_v1_pred_00037810	2A	1.80E-102	348.8	16.6
GrKUP33	D5_v1_pred_00038061	2B	1.60E-165	556.9	20
GrKUP34	D5_v1_pred_00038508	2B	2.10E-189	635.7	23.3
GrKUP35	D5_v1_pred_00038549	1	1.10E-83	286.8	29.5
GaKUP01	Ga01G1031	2C	6.40E-75	257.9	7.8
GaKUP02	Ga01G1612	2B	6.00E-192	644.1	19
GaKUP03	Ga01G1710	2A	5.90E-174	584.7	23.1
GaKUP04	Ga02G0492	2A	2.00E-05	28.5	0.1
GaKUP05	Ga02G1270	1	2.70E-190	638.6	19
GaKUP06	Ga03G2788	3	3.10E-162	546.1	19.8
GaKUP07	Ga04G0059	2B	1.30E-192	646.3	15.1

GaKUP08	Ga04G0192	5	1.00E-167	564.1	25.9
GaKUP09	Ga04G1306	2A	6.60E-191	640.6	26.4
GaKUP10	Ga04G1520	2B	1.80E-188	632.6	21.2
GaKUP11	Ga04G2003	2B	4.40E-194	651.2	20.6
GaKUP12	Ga04G2050	1	4.40E-82	281.5	12.9
GaKUP13	Ga05G2970	2B	2.00E-191	642.4	19.4
GaKUP14	Ga05G3450	4	1.10E-189	636.7	22.3
GaKUP15	Ga05G3567	5	3.30E-186	625.1	28
GaKUP16	Ga08G2359	2C	1.90E-185	622.7	28.9
GaKUP17	Ga09G0320	2B	6.70E-90	307.3	11.9
GaKUP18	Ga09G0321	2B	1.20E-36	131.6	0.2
GaKUP19	Ga10G2580	2C	2.20E-188	632.3	20.5
GaKUP20	Ga11G1031	1	5.00E-174	584.9	21.9
GaKUP21	Ga11G2828	1	2.30E-169	569.5	33.2
GaKUP22	Ga11G3420	2A	6.40E-151	508.7	21.6
GaKUP23	Ga12G0070	2B	1.10E-156	527.8	15.3
GaKUP24	Ga12G2632	3	4.80E-180	604.8	24.4
GaKUP25	Ga12G2769	5	3.00E-169	569.2	29.1
GaKUP26	Ga12G2824	1	8.70E-197	660.1	22.4
GbKUP01	GB_A01G0924	2C	2.10E-148	500.4	17.9
GbKUP02	GB_A01G1518	2B	6.40E-150	505.4	19.9
GbKUP03	GB_A01G1588	4	1.90E-189	635.8	22.3

GbKUP04	GB_A01G2052	1	2.50E-190	638.7	19
GbKUP05	GB_A01G2054	1	2.30E-157	529.9	15.7
GbKUP06	GB_A02G1178	2A	1.70E-174	586.5	23.3
GbKUP07	GB_A03G2523	3	1.90E-116	394.9	8.3
GbKUP08	GB_A04G1412	5	3.70E-168	565.6	26.2
GbKUP09	GB_A04G1684	2B	1.50E-192	646	14.8
GbKUP10	GB_A05G2831	2B	2.20E-192	645.5	19.7
GbKUP11	GB_A05G3480	5	6.50E-186	624.2	27.7
GbKUP12	GB_A05G3561	2A	6.40E-191	640.7	25.9
GbKUP13	GB_A05G3890	2B	1.60E-188	632.8	21.2
GbKUP14	GB_A05G4337	2B	2.20E-193	648.8	21.2
GbKUP15	GB_A05G4373	1	2.40E-50	176.8	8.8
GbKUP16	GB_A08G2399	2C	3.10E-185	621.9	28.3
GbKUP17	GB_A09G0322	2B	1.70E-186	626.1	22.6
GbKUP18	GB_A10G0482	2C	6.20E-190	637.4	21
GbKUP19	GB_A11G1215	1	7.10E-127	429.3	37.9
GbKUP20	GB_A11G2904	1	2.20E-178	599.3	23.2
GbKUP21	GB_A12G0086	1	5.20E-196	657.5	22.7
GbKUP22	GB_A12G0138	5	2.60E-169	569.4	28.9
GbKUP23	GB_A12G0484	3	1.80E-158	533.6	23.4
GbKUP24	GB_A12G3008	2B	1.20E-156	527.6	15.4
GbKUP25	GB_D01G0983	2C	4.00E-156	525.9	24.4

GbKUP26	GB_D01G1649	2B	2.40E-192	645.4	18.7
GbKUP27	GB_D01G1658	5	2.90E-134	453.7	27.3
GbKUP28	GB_D01G1687	4	1.10E-187	630.1	21.6
GbKUP29	GB_D01G2145	1	7.90E-190	637.1	20.4
GbKUP30	GB_D02G1117	5	5.00E-26	96.5	5.8
GbKUP31	GB_D02G2655	3	2.20E-159	536.6	25.1
GbKUP32	GB_D03G0907	2A	1.30E-172	580.2	24.6
GbKUP33	GB_D04G0094	1	3.10E-136	460.2	16.2
GbKUP34	GB_D04G0132	2B	4.40E-194	651.1	20.9
GbKUP35	GB_D04G0571	2B	1.80E-188	632.6	21.2
GbKUP36	GB_D04G0781	2A	1.20E-191	643.1	25.2
GbKUP37	GB_D04G1129	5	9.70E-13	52.7	0.2
GbKUP38	GB_D04G1799	5	2.70E-168	566	25.1
GbKUP39	GB_D05G2819	2B	5.00E-192	644.3	20
GbKUP40	GB_D05G3475	5	3.40E-185	621.8	27.7
GbKUP41	GB_D08G1693	5	5.10E-23	86.6	7.1
GbKUP42	GB_D08G2389	2C	3.70E-185	621.7	29.9
GbKUP43	GB_D09G0294	2B	3.60E-188	631.6	22.1
GbKUP44	GB_D10G0496	2C	2.60E-190	638.7	19.4
GbKUP45	GB_D10G1027	5	5.50E-36	129.4	0.8
GbKUP46	GB_D11G1253	1	8.70E-193	646.9	28.9
GbKUP47	GB_D11G2890	1	1.10E-190	639.9	23.4

GbKUP48	GB_D12G0095	1	3.90E-196	657.9	21.6
GbKUP49	GB_D12G0148	5	2.20E-169	569.6	29.1
GbKUP50	GB_D12G0481	3	1.70E-187	629.4	24.4
GbKUP51	GB_D12G3016	2B	8.00E-133	449	18.7
GhKUP01	GH_A01G0927	2C	7.60E-130	439.1	20.1
GhKUP02	GH_A01G1446	2B	6.40E-150	505.4	19.9
GhKUP03	GH_A01G1525	4	1.90E-189	635.8	22.3
GhKUP04	GH_A01G1955	1	2.50E-190	638.7	19
GhKUP05	GH_A01G1956	1	6.80E-156	525.1	16.3
GhKUP06	GH_A02G1165	2A	2.90E-174	585.7	23.2
GhKUP07	GH_A03G2440	3	6.80E-103	350.2	8.7
GhKUP08	GH_A04G1366	5	3.40E-169	569	27
GhKUP09	GH_A04G1640	2B	3.10E-192	645	14.6
GhKUP10	GH_A05G2795	2B	2.20E-192	645.5	19.7
GhKUP11	GH_A05G3416	5	6.50E-186	624.2	27.7
GhKUP12	GH_A05G3487	2A	7.20E-191	640.5	25.7
GhKUP13	GH_A05G3796	2B	1.80E-188	632.6	21.2
GhKUP14	GH_A05G4240	2B	2.20E-193	648.8	21.2
GhKUP15	GH_A05G4279	1	1.60E-50	177.4	8.5
GhKUP16	GH_A08G2288	2C	3.10E-185	622	28.1
GhKUP17	GH_A09G0295	2B	1.60E-186	626.2	22.7
GhKUP18	GH_A10G0484	2C	1.40E-189	636.3	20.3

GhKUP19	GH_A11G0649	2A	4.40E-164	552.1	19.3
GhKUP20	GH_A11G1210	1	2.10E-149	503.7	35
GhKUP21	GH_A11G2837	1	5.00E-178	598.1	23.9
GhKUP22	GH_A12G0089	1	9.20E-196	656.7	21.5
GhKUP23	GH_A12G0139	5	3.50E-169	569	29.1
GhKUP24	GH_A12G0462	3	2.70E-187	628.7	24.5
GhKUP25	GH_A12G2910	2B	2.60E-156	526.5	15.4
GhKUP26	GH_D01G0944	2C	1.30E-149	504.3	32.4
GhKUP27	GH_D01G1585	2B	4.00E-192	644.7	18.7
GhKUP28	GH_D01G1593	5	1.90E-91	312.4	22.5
GhKUP29	GH_D01G1614	4	7.60E-188	630.6	22.1
GhKUP30	GH_D01G2051	1	1.40E-189	636.2	20.4
GhKUP31	GH_D02G1069	5	9.50E-26	95.6	5.7
GhKUP32	GH_D02G2602	3	2.20E-154	520.1	12.6
GhKUP33	GH_D03G0898	2A	3.70E-174	585.4	24.2
GhKUP34	GH_D04G0097	1	7.60E-173	581	18.3
GhKUP35	GH_D04G0136	2B	3.80E-194	651.3	20.8
GhKUP36	GH_D04G0555	2B	1.80E-188	632.6	21.2
GhKUP37	GH_D04G0753	2A	1.20E-191	643.1	25.2
GhKUP38	GH_D04G1076	5	2.20E-06	31.7	0.1
GhKUP39	GH_D04G1711	5	2.60E-168	566.1	25.1
GhKUP40	GH_D05G2808	2B	9.70E-193	646.7	20

GhKUP41	GH_D05G3342	5	3.40E-185	621.8	27.7
GhKUP42	GH_D05G3458	5	1.30E-39	141.4	2
GhKUP43	GH_D08G1626	5	5.20E-24	89.9	6.9
GhKUP44	GH_D08G2294	2C	3.50E-185	621.8	29.4
GhKUP45	GH_D09G0302	2B	3.60E-188	631.6	22.1
GhKUP46	GH_D10G0510	2C	2.10E-190	639	19
GhKUP47	GH_D10G1035	5	4.30E-36	129.7	0.9
GhKUP48	GH_D11G1242	1	8.70E-193	646.9	28.9
GhKUP49	GH_D11G2869	1	1.40E-191	642.8	23.9
GhKUP50	GH_D12G0094	1	2.20E-197	662	21.8
GhKUP51	GH_D12G0148	5	3.50E-169	569	29
GhKUP52	GH_D12G0476	3	1.70E-187	629.4	24.4
GhKUP53	GH_D12G2933	2B	7.30E-149	501.9	17.4

Table S4 KUP proteins identified in *A. thaliana*, *B. ceiba*, *C. capsularis*, *C. olitorius*, *D. zibethinus*, *G. australe*, *G. kirkii*, *G. turneri*, *H. umbratica*, *T. cacao*, *V. vinifera*, and *O. sativa*.

Name	Locus name	Subfamily	E-value	Score	Bias
AtKUP01	AT1G31120	3	3.80E-187	628.2	23.1
AtKUP02	AT1G60160	5	9.10E-184	617.1	27.7
AtKUP03	AT1G70300	2B	2.20E-188	632.3	23.1
AtKUP04	AT2G30070	2C	1.30E-185	623.2	25
AtKUP05	AT2G35060	3	1.30E-183	616.6	23.4
AtKUP06	AT2G40540	2B	8.70E-193	646.9	17.5
AtKUP07	AT3G02050	2A	2.90E-182	612.1	24.4
AtKUP08	AT4G13420	1	1.20E-191	643.1	32.2
AtKUP09	AT4G19960	3	1.30E-176	593.5	24
AtKUP10	AT4G23640	2A	1.20E-170	573.8	26.2
AtKUP11	AT4G33530	5	7.30E-165	554.7	28.7
AtKUP12	AT5G09400	5	1.20E-164	554	28.3
AtKUP13	AT5G14880	2B	3.80E-191	641.5	21.5
BcKUP01	evm.model.Scaffold101.69	2B	2.50E-192	645.4	20.8
BcKUP02	evm.model.Scaffold106.763	3	2.10E-185	622.5	23.7
BcKUP03	evm.model.Scaffold11.528	1	9.40E-181	607.2	12.9
BcKUP04	evm.model.Scaffold11.529	1	8.10E-148	498.5	20.8
BcKUP05	evm.model.Scaffold11.531	1	7.90E-182	610.7	22.3
BcKUP06	evm.model.Scaffold142.178	2B	1.90E-130	441.1	2.3

BcKUP07	evm.model.Scaffold142.3	2A	2.30E-11	48.1	0
BcKUP08	evm.model.Scaffold145.40	2C	1.40E-176	593.4	29.1
BcKUP09	evm.model.Scaffold1666.5	1	4.30E-183	614.9	22.4
BcKUP10	evm.model.Scaffold1666.6	1	2.50E-180	605.7	23.8
BcKUP11	evm.model.Scaffold174.130	2B	1.60E-190	639.4	19.7
BcKUP12	evm.model.Scaffold19.3	2B	9.50E-192	643.4	19.6
BcKUP13	evm.model.Scaffold193.15	1	2.00E-189	635.8	22
BcKUP14	evm.model.Scaffold228.80	2C	6.50E-71	244.7	21.2
BcKUP15	evm.model.Scaffold258.651	1	1.00E-184	620.2	24.6
BcKUP16	evm.model.Scaffold287.10	2B	9.00E-151	508.2	18.3
BcKUP17	evm.model.Scaffold305.11	2B	1.10E-190	639.9	19.5
BcKUP18	evm.model.Scaffold305.12	5	8.30E-185	620.5	28.2
BcKUP19	evm.model.Scaffold410.25	2B	7.20E-194	650.4	20.1
BcKUP20	evm.model.Scaffold431.27	2B	8.10E-11	46.3	0
BcKUP21	evm.model.Scaffold47.164	2A	2.20E-177	596	26.1
BcKUP22	evm.model.Scaffold48.310	4	2.80E-182	612.2	19.7
BcKUP23	evm.model.Scaffold485.20	5	2.20E-156	526.7	27.6
BcKUP24	evm.model.Scaffold6.31	5	1.10E-181	610.3	30.4
BcKUP25	evm.model.Scaffold64.384	2A	1.20E-30	111.8	6.2
BcKUP26	evm.model.Scaffold64.385	2A	4.10E-21	80.3	0
BcKUP27	evm.model.Scaffold648.13	5	3.30E-168	565.7	30.3
BcKUP28	evm.model.Scaffold72.486	3	4.70E-187	628	24.3

BcKUP29	evm.model.Scaffold8.404	2C	1.40E-175	590.1	18.1
BcKUP30	evm.model.Scaffold850.1	2B	3.70E-182	611.8	22.1
BcKUP31	evm.model.Scaffold89.316	2A	1.00E-128	435.4	19.3
BcKUP32	evm.model.Scaffold91.9	2B	1.80E-136	461	6.8
CcKUP01	OMO60781	4	1.80E-188	632.6	21.1
CcKUP02	OMO63658	2B	1.40E-91	312.9	5.1
CcKUP03	OMO78177	2B	5.60E-192	644.2	17.7
CcKUP04	OMO78191	5	2.40E-187	628.9	29.2
CcKUP05	OMO79640	2A	2.20E-63	219.8	10.4
CcKUP06	OMO85599	2A	2.50E-97	331.8	13.5
CcKUP07	OMO86998	5	1.40E-169	570.3	27.6
CcKUP08	OMO88353	2C	4.90E-175	588.3	25
CcKUP09	OMO88829	2B	1.00E-163	550.9	16.5
CoKUP01	COL.rna12183	2A	5.50E-85	291.1	0.1
CoKUP02	COL.rna12549	2C	4.20E-08	37.4	0.4
CoKUP03	COL.rna15451	1	7.30E-186	624	23.1
CoKUP04	COL.rna16408	3	2.20E-131	444.2	15.7
CoKUP05	COL.rna18214	2B	6.90E-196	657.1	18.1
CoKUP06	COL.rna18282	2B	3.90E-192	644.7	18.2
CoKUP07	COL.rna22143	1	1.50E-182	613.1	22.1
CoKUP08	COL.rna22144	1	2.50E-153	516.6	15.6
CoKUP09	COL.rna22145	1	7.80E-102	346.7	9.5

CoKUP10	COL.rna23676	5	3.10E-170	572.4	28.1
CoKUP11	COL.rna2527	1	8.00E-178	597.5	24.6
CoKUP12	COL.rna26170	1	4.80E-36	129.6	0.2
CoKUP13	COL.rna26171	1	2.90E-115	391	10.8
CoKUP14	COL.rna26280	4	3.10E-133	450.3	14.8
CoKUP15	COL.rna27643	3	1.30E-145	491.2	14.5
CoKUP16	COL.rna29638	2C	7.60E-173	581.1	21.1
CoKUP17	COL.rna31414	2B	6.80E-161	541.6	13.2
CoKUP18	COL.rna33333	1	9.10E-184	617.1	23.6
CoKUP19	COL.rna339	1	2.70E-162	546.2	24.5
CoKUP20	COL.rna35742	2B	9.00E-185	620.4	23
CoKUP21	COL.rna37038	2C	2.00E-185	622.6	27.5
CoKUP22	COL.rna6796	1	9.10E-37	132	0.3
CoKUP23	COL.rna7139	2A	2.30E-56	196.6	11.9
CoKUP24	COL.rna7911	1	1.40E-126	428.4	12.3
CoKUP25	COL.rna7912	1	1.50E-10	45.4	0
CoKUP26	COL.rna9888	2C	4.10E-153	515.9	27.7
DzKUP01	XP_022715410	2C	2.70E-190	638.6	19.1
DzKUP02	XP_022718099	2C	5.40E-179	601.3	24
DzKUP03	XP_022723695	2C	1.40E-191	642.9	17.9
DzKUP04	XP_022726896	2B	2.30E-190	638.9	16
DzKUP05	XP_022727086	3	2.30E-184	619.1	23.7

DzKUP06	XP_022729350	2C	8.40E-185	620.5	29.7
DzKUP07	XP_022732787	1	1.00E-196	659.8	23
DzKUP08	XP_022733943	4	5.70E-191	640.9	20.5
DzKUP09	XP_022733947	5	1.50E-183	616.3	28
DzKUP10	XP_022734887	2B	1.30E-191	643	18.7
DzKUP11	XP_022737659	2A	6.20E-173	581.3	23.6
DzKUP12	XP_022739218	2A	6.00E-191	640.8	24.9
DzKUP13	XP_022740293	2B	2.50E-189	635.5	21.1
DzKUP14	XP_022747179	2B	1.00E-193	649.9	19.4
DzKUP15	XP_022748751	3	1.90E-188	632.5	22.2
DzKUP16	XP_022748918	1	5.70E-193	647.5	20.2
DzKUP17	XP_022750419	2A	7.80E-190	637.1	25.8
DzKUP18	XP_022758030	1	1.90E-197	662.2	23.1
DzKUP19	XP_022758031	1	1.60E-192	646	24.1
DzKUP20	XP_022760024	1	1.00E-196	659.8	23
DzKUP21	XP_022762625	3	1.20E-187	629.8	23
DzKUP22	XP_022763694	5	4.70E-167	561.9	30.2
DzKUP23	XP_022764868	2B	3.60E-193	648.1	19.3
DzKUP24	XP_022769813	1	9.50E-194	650	21.9
DzKUP25	XP_022774178	2A	1.10E-189	636.6	25.8
DzKUP26	XP_022776066	2B	1.10E-190	639.9	19.5
GauKUP01	KAA3453227	5	9.80E-185	620.3	28.4

GauKUP02	KAA3453512	2B	2.00E-192	645.7	19.7
GauKUP03	KAA3455962	2B	9.80E-189	633.5	21.4
GauKUP04	KAA3457067	2A	5.70E-72	248.2	8.8
GauKUP05	KAA3459713	2B	7.80E-37	132.2	0
GauKUP06	KAA3460990	3	1.50E-181	609.7	24.3
GauKUP07	KAA3463026	5	6.30E-169	568.1	29.1
GauKUP08	KAA3463070	1	5.10E-196	657.5	22.2
GauKUP09	KAA3463380	2B	6.50E-191	640.7	15.4
GauKUP10	KAA3463575	5	2.30E-168	566.3	24.9
GauKUP11	KAA3464199	2A	6.40E-191	640.7	25.9
GauKUP12	KAA3464600	2B	1.20E-193	649.7	20.7
GauKUP13	KAA3465371	1	3.80E-189	634.9	20.3
GauKUP14	KAA3465376	1	7.10E-191	640.6	18.4
GauKUP15	KAA3466120	2B	1.70E-180	606.3	17.7
GauKUP16	KAA3466303	4	1.30E-179	603.4	20.6
GauKUP17	KAA3468756	2C	9.90E-164	551	29.1
GauKUP18	KAA3472871	1	3.10E-191	641.7	24.1
GauKUP19	KAA3475060	1	9.10E-192	643.5	28.1
GauKUP20	KAA3475519	2A	8.20E-182	610.6	24.7
GauKUP21	KAA3479127	2C	2.10E-98	335.4	6
GkKUP01	Kirkii_Version3_Juiced.00g016920	5	2.10E-83	285.9	23.9
GkKUP02	Kirkii_Version3_Juiced.00g017140	4	2.90E-188	631.9	24.2

GkKUP03	Kirkii_Version3_Juiced.00g021660	1	1.00E-190	640	20.3
GkKUP04	Kirkii_Version3_Juiced.00g021670	1	7.20E-190	637.2	19.9
GkKUP05	Kirkii_Version3_Juiced.00g029260	2B	8.90E-32	115.5	0
GkKUP06	Kirkii_Version3_Juiced.00g032390	5	2.70E-161	542.9	23.3
GkKUP07	Kirkii_Version3_Juiced.00g081000	3	4.50E-16	63.7	0.1
GkKUP08	Kirkii_Version3_Juiced.00g081010	3	1.60E-96	329.1	24.2
GkKUP09	Kirkii_Version3_Juiced.00g081030	3	4.80E-27	99.9	0.2
GkKUP10	Kirkii_Version3_Juiced.00g081040	3	5.30E-72	248.3	8.4
GkKUP11	Kirkii_Version3_Juiced.00g081070	3	2.40E-26	97.6	0.3
GkKUP12	Kirkii_Version3_Juiced.00g112760	2B	2.30E-12	51.4	0
GkKUP13	Kirkii_Version3_Juiced.00g145570	2B	0.00012	25.9	0
GkKUP14	Kirkii_Version3_Juiced.00g145580	2B	1.60E-08	38.7	0
GkKUP15	Kirkii_Version3_Juiced.00g154940	2B	7.90E-189	633.8	20.7
GkKUP16	Kirkii_Version3_Juiced.00g167810	2A	1.80E-34	124.4	0.1
GkKUP17	Kirkii_Version3_Juiced.00g229740	2B	8.00E-97	330.2	13.1
GkKUP18	Kirkii_Version3_Juiced.00g229750	2B	3.10E-41	146.7	0.2
GkKUP19	Kirkii_Version3_Juiced.00g279910	2C	5.60E-180	604.6	17.6
GkKUP20	Kirkii_Version3_Juiced.00g299230	1	1.60E-21	81.7	0.6
GkKUP21	Kirkii_Version3_Juiced.00g299240	1	6.00E-158	531.9	24.7
GkKUP22	Kirkii_Version3_Juiced.00g316520	1	1.10E-142	481.5	20.5
GkKUP23	Kirkii_Version3_Juiced.00g328260	2B	5.00E-81	278	7.2
GkKUP24	Kirkii_Version3_Juiced.00g355210	1	2.70E-78	269	9.8

GkKUP25	Kirkii_Version3_Juiced.00g355220	1	2.30E-23	87.7	0.6
GkKUP26	Kirkii_Version3_Juiced.00g375070	1	8.60E-28	102.3	13
GkKUP27	Kirkii_Version3_Juiced.00g375090	1	2.50E-46	163.5	1.3
GkKUP28	Kirkii_Version3_Juiced.00g375100	1	4.10E-157	529.1	19.1
GkKUP29	Kirkii_Version3_Juiced.00g375110	1	6.90E-06	30.1	0.1
GkKUP30	Kirkii_Version3_Juiced.00g375120	1	1.90E-21	81.4	1.2
GtKUP01	TURN.00g284830	5	1.20E-07	35.9	3.1
GtKUP02	TURN.00g290270	2C	4.20E-190	638	19.3
GtKUP03	TURN.01g006120	1	1.80E-150	507.2	14.2
GtKUP04	TURN.01g006140	1	7.70E-12	49.7	0
GtKUP05	TURN.01g006150	1	2.80E-189	635.3	20
GtKUP06	TURN.01g011100	4	3.10E-175	588.9	24.9
GtKUP07	TURN.01g011380	5	4.70E-56	195.6	22.9
GtKUP08	TURN.01g011480	2B	6.30E-48	168.8	0
GtKUP09	TURN.01g011490	2B	3.90E-92	314.7	8.7
GtKUP10	TURN.01g018040	2C	6.30E-13	53.3	0
GtKUP11	TURN.01g018050	2C	7.90E-131	442.4	12.5
GtKUP12	TURN.01g302440	2A	1.70E-157	530.4	15.7
GtKUP13	TURN.01g308650	1	1.90E-192	645.7	28.8
GtKUP14	TURN.01g325510	1	7.90E-192	643.7	23.2
GtKUP15	TURN.02g036350	5	2.20E-05	28.4	1
GtKUP16	TURN.02g038680	2A	8.60E-175	587.5	23.3

GtKUP17	TURN.02g336290	1	2.60E-197	661.8	22
GtKUP18	TURN.02g336910	5	2.40E-169	569.5	28.8
GtKUP19	TURN.02g339690	3	3.20E-187	628.5	24.2
GtKUP20	TURN.02g365250	2B	2.30E-153	516.8	15.8
GtKUP21	TURN.03g050620	3	5.90E-46	162.3	7.4
GtKUP22	TURN.03g050630	3	5.60E-61	211.9	3.3
GtKUP23	TURN.04g081140	1	1.80E-11	48.5	0.1
GtKUP24	TURN.04g081150	1	4.10E-113	383.9	16.1
GtKUP25	TURN.04g081580	2B	7.90E-183	614	20.2
GtKUP26	TURN.04g088960	2A	1.10E-144	488.1	25.8
GtKUP27	TURN.04g092370	5	8.30E-26	95.8	6.6
GtKUP28	TURN.04g099430	5	3.60E-168	565.6	25.1
GtKUP29	TURN.04g102790	2B	6.60E-97	330.5	15.2
GtKUP30	TURN.05g112450	5	1.00E-24	92.2	0.2
GtKUP31	TURN.05g113240	5	1.70E-185	622.8	27.8
GtKUP32	TURN.08g211080	2C	2.30E-185	622.3	29.2
GtKUP33	TURN.09g260690	2B	2.40E-176	592.6	24
HuKUP01	XM_021417944	5	8.20E-167	561.1	28.6
HuKUP02	XM_021423792	2C	2.90E-184	618.7	31.5
HuKUP03	XM_021424106	1	2.10E-180	606	23.5
HuKUP04	XM_021425275	1	2.10E-192	645.6	24.7
HuKUP05	XM_021425300	1	1.50E-138	467.9	13

HuKUP06	XM_021428473	1	2.60E-192	645.3	21.6
HuKUP07	XM_021432347	2C	3.40E-187	628.4	19.7
HuKUP08	XM_021436814	2B	2.00E-147	497.2	16.3
HuKUP09	XM_021436997	5	3.20E-185	621.9	28.2
HuKUP10	XM_021437216	4	2.60E-186	625.5	21.9
HuKUP11	XM_021437846	2B	7.90E-191	640.4	19.6
HuKUP12	XM_021438365	2C	2.30E-192	645.5	20.1
HuKUP13	XM_021441194	2B	1.70E-193	649.2	18.5
HuKUP14	XM_021442160	2A	2.60E-189	635.4	25.1
HuKUP15	XM_021443604	2B	7.40E-189	633.9	22.4
HuKUP16	XM_021443700	2A	1.80E-172	579.8	21.9
HuKUP17	XM_021444535	3	1.20E-186	626.6	24.3
TcKUP01	Thecc01G281300	1	2.10E-42	150.6	1.4
TcKUP02	Thecc01G294600	1	3.60E-107	364.3	26
TcKUP03	Thecc01G294700	1	1.20E-193	649.7	23.9
TcKUP04	Thecc01G294800	1	2.60E-174	585.9	23.5
TcKUP05	Thecc02G007000	3	1.20E-186	626.7	25
TcKUP06	Thecc02G138800	5	1.50E-167	563.6	28.3
TcKUP07	Thecc02G189200	1	2.00E-191	642.4	18.5
TcKUP08	Thecc02G245500	5	1.10E-185	623.5	27
TcKUP09	Thecc02G245600	2B	3.30E-191	641.6	19.6
TcKUP10	Thecc02G301300	4	2.90E-187	628.6	20.9

TcKUP11	Thecc03G061800	2B	4.10E-62	215.6	4
TcKUP12	Thecc03G288900	2A	1.30E-173	583.6	23.1
TcKUP13	Thecc04G007000	2B	4.70E-189	634.5	21.6
TcKUP14	Thecc04G050400	2A	4.10E-191	641.3	26.4
TcKUP15	Thecc06G135100	2C	5.10E-192	644.3	19.7
TcKUP16	Thecc07G160700	2C	5.50E-186	624.4	20.7
TcKUP17	Thecc09G351800	2C	4.40E-185	621.4	31.3
TcKUP18	Thecc10G183700	2B	1.20E-193	649.8	19.3
VvKUP01	VIT_200s0125g00190	2C	1.80E-189	635.9	23.7
VvKUP02	VIT_201s0011g01510	3	1.20E-186	626.6	23.2
VvKUP03	VIT_201s0011g03020	1	7.40E-193	647.1	19.7
VvKUP04	VIT_201s0011g06560	4	1.20E-189	636.5	18.4
VvKUP05	VIT_201s0026g00260	5	1.90E-185	622.7	24.9
VvKUP06	VIT_201s0026g00270	2B	5.60E-190	637.6	18
VvKUP07	VIT_202s0025g00680	2A	1.30E-126	428.5	22.5
VvKUP08	VIT_202s0025g03820	2B	4.40E-17	67	0
VvKUP09	VIT_202s0025g04862	2C	1.80E-15	61.7	1.3
VvKUP10	VIT_206s0004g06890	2C	2.00E-186	625.9	30.4
VvKUP11	VIT_207s0005g06410	2C	8.80E-34	122.1	2
VvKUP12	VIT_207s0104g01650	1	4.90E-25	93.3	0.1
VvKUP13	VIT_207s0104g01660	1	4.00E-191	641.4	23.4
VvKUP14	VIT_207s0104g01670	1	2.20E-18	71.3	0.3

VvKUP15	VIT_207s0104g01710	1	1.40E-193	649.4	26.3
VvKUP16	VIT_207s0104g01720	1	1.20E-190	639.9	23.1
VvKUP17	VIT_207s0104g01730	1	6.20E-195	654	21.7
VvKUP18	VIT_212s0028g00380	2C	2.70E-07	34.7	0.6
VvKUP19	VIT_212s0142g00170	5	3.10E-119	404.2	24.9
VvKUP20	VIT_212s0142g00250	5	1.30E-41	148	1.4
VvKUP21	VIT_213s0067g02570	2B	4.40E-195	654.5	17.7
VvKUP22	VIT_213s0139g00390	2B	2.60E-20	77.7	0.8
VvKUP23	VIT_213s0139g00410	2B	5.30E-06	30.5	0.1
VvKUP24	VIT_214s0066g01580	2B	9.50E-186	623.6	16.8
VvKUP25	VIT_214s0068g00850	2A	8.50E-188	630.4	24.1
VvKUP26	VIT_217s0000g01930	2B	1.80E-156	527	20.5
VvKUP27	VIT_217s0000g10435	4	6.70E-15	59.8	1.2
VvKUP28	VIT_217s0000g10445	4	1.80E-17	68.3	0
VvKUP29	VIT_219s0015g02820	2C	2.00E-05	28.5	0.1
VvKUP30	VIT_219s0027g01820	2C	9.30E-154	518.1	25
VvKUP31	VIT_219s0093g00170	2B	0.00016	25.6	2.3
OsKUP01	LOC_Os01g27170	2A	8.70E-183	613.9	19.8
OsKUP02	LOC_Os01g70490	1	3.30E-175	588.8	26.3
OsKUP03	LOC_Os01g70660	4	1.40E-192	646.2	17.4
OsKUP04	LOC_Os01g70940	2A	8.40E-189	633.7	22.2
OsKUP05	LOC_Os02g31910	1	2.10E-190	639	17

OsKUP06	LOC_Os02g31940	1	2.70E-189	635.3	16.7
OsKUP07	LOC_Os02g49760	2B	2.20E-188	632.3	23.6
OsKUP08	LOC_Os03g21890	2B	6.30E-193	647.3	16.8
OsKUP09	LOC_Os03g37830	1	5.40E-174	584.8	18.5
OsKUP10	LOC_Os03g37840	1	1.60E-168	566.8	18.6
OsKUP11	LOC_Os03g37930	1	7.10E-174	584.5	26.1
OsKUP12	LOC_Os03g55370	2B	1.00E-06	32.8	0.1
OsKUP13	LOC_Os04g32920	1	2.50E-187	628.9	20.9
OsKUP14	LOC_Os04g52120	5	1.40E-153	517.5	25.9
OsKUP15	LOC_Os04g52390	3	2.10E-182	612.6	26.3
OsKUP16	LOC_Os06g15910	2B	4.30E-187	628.1	21.4
OsKUP17	LOC_Os06g42030	2B	1.10E-193	649.9	13.1
OsKUP18	LOC_Os06g45940	2C	2.30E-186	625.7	14.4
OsKUP19	LOC_Os07g32530	5	7.50E-166	558	31.7
OsKUP20	LOC_Os07g47350	2A	2.10E-188	632.4	21.7
OsKUP21	LOC_Os07g48130	2B	8.40E-195	653.5	19.3
OsKUP22	LOC_Os08g10550	3	1.40E-131	444.9	27.8
OsKUP23	LOC_Os08g36340	4	2.00E-180	606.1	14.7
OsKUP24	LOC_Os08g39950	4	4.50E-190	637.9	31.3
OsKUP25	LOC_Os09g21000	5	2.00E-182	612.7	30.9
OsKUP26	LOC_Os09g27580	4	4.20E-186	624.8	20.9
OsKUP27	LOC_Os09g38960	3	8.30E-179	600.7	27.9

Table S5 Locations of the conserved KUP domain in the KUP members from the 13 Malvaceae species, *T. cacao*, *V. vinifera*, and *O. sativa*.

Name	Protein length (aa)	Start (aa)	End (aa)	E-Value	Bitscore
AtKUP01	797	22	796	0	900.781
AtKUP02	828	1	827	0	1574.36
AtKUP03	783	1	782	0	1464.3
AtKUP04	713	28	712	0	1001.22
AtKUP05	794	23	793	0	893.847
AtKUP06	795	15	794	0	1135.34
AtKUP07	790	2	789	0	1500.1
AtKUP08	786	2	783	0	863.282
AtKUP09	824	16	823	0	846.082
AtKUP10	776	12	775	0	1089.09
AtKUP11	856	4	855	0	1565.89
AtKUP12	859	7	858	0	1612.88
AtKUP13	782	1	781	0	1475.47
BcKUP01	796	1	796	0	1149.59
BcKUP02	795	13	795	0	940.071
BcKUP03	734	9	707	0	761.589
BcKUP04	641	3	639	0	674.534
BcKUP05	705	37	647	0	757.737
BcKUP06	694	1	661	0	1102.6

BcKUP07	130	1	130	2.36E-78	245.89
BcKUP08	728	6	728	0	1048.65
BcKUP09	758	9	740	0	794.331
BcKUP10	775	37	773	0	800.88
BcKUP11	794	2	794	0	1445.04
BcKUP12	778	1	778	0	1552.51
BcKUP13	809	31	807	0	857.889
BcKUP14	495	70	495	########	344.886
BcKUP15	768	22	766	0	745.796
BcKUP16	784	18	784	0	831.393
BcKUP17	780	1	780	0	1500.9
BcKUP18	846	1	846	0	1580.91
BcKUP19	931	1	792	0	1184.65
BcKUP20	179	41	174	8.92E-49	168.106
BcKUP21	793	1	793	0	1485.08
BcKUP22	732	59	626	0	666.515
BcKUP23	828	22	828	0	1483.45
BcKUP24	844	1	844	0	1535.07
BcKUP25	154	2	128	6.33E-51	172.702
BcKUP26	144	13	125	4.11E-57	189.266
BcKUP27	858	21	858	0	1641.77
BcKUP28	791	13	791	0	950.857

BcKUP29	729	1	729	0	942.331
BcKUP30	802	1	802	0	1519.39
BcKUP31	711	27	711	0	1012.44
BcKUP32	768	1	766	0	778.236
CcKUP01	733	59	627	0	673.834
CcKUP02	638	1	491	0	656.539
CcKUP03	779	1	779	0	1560.99
CcKUP04	846	1	846	0	1552.4
CcKUP05	1577	1	494	0	664.987
CcKUP06	751	3	518	0	961.206
CcKUP07	853	22	853	0	1631.37
CcKUP08	685	10	685	0	876.077
CcKUP09	764	3	764	0	881.084
CoKUP01	552	1	551	0	1005.89
CoKUP02	145	1	74	8.48E-12	61.3794
CoKUP03	817	38	814	0	845.178
CoKUP04	712	15	686	0	720.122
CoKUP05	794	1	793	0	1171.55
CoKUP06	780	1	779	0	1540.57
CoKUP07	751	27	748	0	785.086
CoKUP08	683	28	680	0	676.845
CoKUP09	375	5	374	########	467.297

CoKUP10	867	22	800	0	1503.87
CoKUP11	698	18	695	0	778.923
CoKUP12	226	38	221	2.02E-60	202.665
CoKUP13	522	6	519	0	540.1
CoKUP14	517	59	445	########	470.449
CoKUP15	692	15	691	0	816.422
CoKUP16	772	1	771	0	927.308
CoKUP17	738	2	731	0	802.118
CoKUP18	641	53	639	0	720.373
CoKUP19	760	15	757	0	741.174
CoKUP20	791	1	790	0	1475.09
CoKUP21	703	5	702	0	917.678
CoKUP22	299	39	296	1.03E-47	170.057
CoKUP22	299	2	73	1.49E-22	97.2543
CoKUP23	1312	527	763	########	358.368
CoKUP23	1312	765	975	2.18E-33	139.241
CoKUP24	442	7	432	########	507.743
CoKUP25	226	5	223	2.26E-52	180.072
CoKUP26	702	29	701	0	959.28
DzKUP01	744	1	744	0	948.109
DzKUP02	740	3	740	0	914.211
DzKUP03	745	1	745	0	964.672

DzKUP04	778	1	778	0	1512.45
DzKUP05	774	16	774	0	914.263
DzKUP06	759	28	759	0	1057.12
DzKUP07	780	11	778	0	836.703
DzKUP08	725	57	624	0	686.546
DzKUP09	844	1	844	0	1568.58
DzKUP10	772	1	772	0	1496.27
DzKUP11	776	13	776	0	1151.49
DzKUP12	791	25	791	0	1544.4
DzKUP13	794	2	794	0	1462.76
DzKUP14	793	1	793	0	1171.17
DzKUP15	797	11	797	0	958.946
DzKUP16	806	31	804	0	871.371
DzKUP17	793	1	793	0	1538.23
DzKUP18	786	3	784	0	814.747
DzKUP19	767	48	765	0	792.405
DzKUP20	780	1	778	0	835.933
DzKUP21	784	13	784	0	948.931
DzKUP22	858	21	858	0	1626.75
DzKUP23	793	1	793	0	1171.94
DzKUP24	772	36	770	0	823.606
DzKUP25	784	1	784	0	1553.64

DzKUP26	779	1	779	0	1531.33
GaKUP01	565	11	565	########	387.258
GaKUP02	779	1	779	0	1530.94
GaKUP03	776	12	776	0	1124.53
GaKUP04	56	7	47	2.61E-19	78.7134
GaKUP05	766	19	764	0	848.259
GaKUP06	774	16	774	0	838.378
GaKUP07	778	1	778	0	1495.89
GaKUP08	856	19	856	0	1568.58
GaKUP09	788	1	788	0	1507.42
GaKUP10	777	1	777	0	1456.6
GaKUP11	792	1	792	0	1152.29
GaKUP12	547	72	502	########	446.496
GaKUP13	811	16	811	0	1152.29
GaKUP14	730	58	625	0	681.538
GaKUP15	804	23	804	0	1496.16
GaKUP16	755	28	755	0	1078.31
GaKUP17	478	6	478	0	854.917
GaKUP18	199	30	185	2.22E-97	298.689
GaKUP19	742	1	742	0	967.754
GaKUP20	739	20	737	0	738.862
GaKUP21	719	21	717	0	749.263

GaKUP22	939	7	662	0	1169.98
GaKUP23	791	22	791	0	808.667
GaKUP24	792	15	792	0	933.137
GaKUP25	858	17	858	0	1626.36
GaKUP26	775	44	773	0	815.902
GauKUP01	861	1	842	0	1520.05
GauKUP02	796	1	796	0	1153.83
GauKUP03	764	5	764	0	1406.91
GauKUP04	477	20	477	0	637.253
GauKUP05	202	5	184	7.30E-54	183.129
GauKUP06	817	15	817	0	936.989
GauKUP07	858	23	858	0	1624.05
GauKUP08	765	43	763	0	810.51
GauKUP09	781	1	781	0	1501.28
GauKUP10	856	19	856	0	1560.11
GauKUP11	788	1	788	0	1509.73
GauKUP12	841	62	841	0	1150.75
GauKUP13	842	95	840	0	861.356
GauKUP14	816	39	814	0	866.364
GauKUP15	815	1	815	0	1501.28
GauKUP16	752	89	647	0	663.819
GauKUP17	795	73	795	0	1009.36

GauKUP18	773	7	771	0	791.635
GauKUP19	773	34	771	0	819.369
GauKUP20	786	1	786	0	1351.8
GauKUP21	418	1	416	########	411.911
GbKUP01	677	9	677	0	750.116
GbKUP02	616	1	616	0	1230.1
GbKUP03	730	58	625	0	681.153
GbKUP04	816	39	814	0	864.052
GbKUP05	782	32	780	0	751.189
GbKUP06	1121	12	769	0	1111.82
GbKUP07	406	62	406	########	454.2
GbKUP08	813	7	813	0	1520.05
GbKUP09	778	1	778	0	1497.43
GbKUP10	796	1	796	0	1158.45
GbKUP11	842	1	842	0	1548.17
GbKUP12	788	1	788	0	1509.34
GbKUP13	777	1	777	0	1450.82
GbKUP14	792	1	792	0	1161.54
GbKUP15	519	66	517	2.17E-94	305.128
GbKUP16	755	28	755	0	1077.15
GbKUP17	764	7	764	0	1398.82
GbKUP18	730	5	730	0	955.813

GbKUP19	617	34	615	0	546.263
GbKUP20	748	49	746	0	733.47
GbKUP21	775	44	773	0	829.77
GbKUP22	858	17	858	0	1622.51
GbKUP23	716	15	579	0	686.224
GbKUP24	791	22	791	0	810.978
GbKUP25	573	11	573	0	702.737
GbKUP26	779	1	779	0	1551.74
GbKUP27	775	6	718	0	1117.9
GbKUP28	730	58	625	0	672.678
GbKUP29	809	32	807	0	858.274
GbKUP30	256	60	182	1.07E-66	221.154
GbKUP31	531	16	512	0	586.458
GbKUP32	776	12	776	0	1119.91
GbKUP33	633	3	587	0	620.606
GbKUP34	792	1	792	0	1161.15
GbKUP35	778	1	778	0	1450.05
GbKUP36	788	1	788	0	1508.19
GbKUP37	154	3	80	1.08E-43	152.974
GbKUP38	856	19	856	0	1568.58
GbKUP39	825	30	825	0	1152.68
GbKUP40	842	1	842	0	1547.4

GbKUP41	207	83	205	3.06E-65	214.991
GbKUP42	755	28	755	0	1067.91
GbKUP43	764	5	764	0	1401.52
GbKUP44	730	5	730	0	955.042
GbKUP45	276	83	276	########	377.16
GbKUP46	773	34	771	0	826.688
GbKUP47	774	7	772	0	796.642
GbKUP48	771	19	769	0	820.91
GbKUP49	858	17	858	0	1627.13
GbKUP50	795	15	795	0	947.005
GbKUP51	705	22	705	0	696.959
GhKUP01	658	9	658	0	708.13
GhKUP02	616	1	616	0	1230.1
GhKUP03	730	58	625	0	681.153
GhKUP04	816	39	814	0	864.052
GhKUP05	788	32	786	0	747.337
GhKUP06	776	12	776	0	1119.52
GhKUP07	355	41	355	########	384.479
GhKUP08	856	19	856	0	1554.72
GhKUP09	778	1	778	0	1498.97
GhKUP10	796	1	796	0	1154.99
GhKUP11	841	1	841	0	1548.17

GhKUP12	788	1	788	0	1507.8
GhKUP13	777	1	777	0	1453.13
GhKUP14	792	1	792	0	1161.54
GhKUP15	524	66	522	2.37E-95	307.824
GhKUP16	755	28	755	0	1077.54
GhKUP17	764	7	764	0	1394.97
GhKUP18	730	5	730	0	958.894
GhKUP19	759	12	759	0	1314.82
GhKUP20	717	34	715	0	684.549
GhKUP21	768	7	766	0	760.819
GhKUP22	775	44	773	0	809.354
GhKUP23	858	17	858	0	1622.13
GhKUP24	795	15	795	0	948.16
GhKUP25	791	22	791	0	806.355
GhKUP26	604	11	604	0	691.181
GhKUP27	779	1	779	0	1547.89
GhKUP28	644	1	587	0	859.815
GhKUP29	730	58	625	0	672.293
GhKUP30	817	2	815	0	860.586
GhKUP31	256	60	182	1.32E-67	223.465
GhKUP32	743	16	743	0	827.208
GhKUP33	776	12	776	0	1116.83

GhKUP34	692	3	646	0	728.847
GhKUP35	792	1	792	0	1161.92
GhKUP36	777	1	777	0	1454.67
GhKUP37	788	1	788	0	1509.34
GhKUP38	130	2	56	4.79E-31	115.61
GhKUP39	856	19	856	0	1560.11
GhKUP40	825	30	825	0	1153.06
GhKUP41	842	1	842	0	1547.4
GhKUP42	292	83	289	########	402.968
GhKUP43	207	83	205	3.74E-66	217.687
GhKUP44	755	28	755	0	1070.6
GhKUP45	764	5	764	0	1401.52
GhKUP46	730	5	730	0	948.879
GhKUP47	276	83	276	########	379.471
GhKUP48	773	34	771	0	826.688
GhKUP49	773	7	771	0	801.265
GhKUP50	771	19	769	0	822.451
GhKUP51	858	17	858	0	1624.44
GhKUP52	795	15	795	0	948.931
GhKUP53	746	3	746	0	805.2
GkKUP01	466	1	460	0	655.274
GkKUP02	728	58	625	0	678.071

GkKUP03	809	32	807	0	868.29
GkKUP04	806	29	804	0	839.014
GkKUP05	340	1	340	0	636.894
GkKUP06	1070	6	869	0	1513.5
GkKUP07	140	51	138	1.20E-23	94.8088
GkKUP08	496	89	377	########	352.507
GkKUP09	139	16	135	1.08E-45	157.731
GkKUP10	491	89	302	9.89E-92	297.039
GkKUP11	146	16	142	1.02E-48	166.205
GkKUP12	298	1	282	3.52E-96	299.818
GkKUP13	78	18	57	1.98E-15	68.6982
GkKUP14	282	14	282	6.30E-94	293.27
GkKUP15	823	1	778	0	1450.44
GkKUP16	301	1	301	0	569.072
GkKUP17	484	1	484	0	868.784
GkKUP18	181	5	170	2.44E-97	297.919
GkKUP19	777	1	777	0	941.175
GkKUP20	134	34	126	5.05E-33	121.388
GkKUP21	648	7	646	0	671.067
GkKUP22	556	53	456	########	521.995
GkKUP22	556	497	554	3.06E-10	62.9716
GkKUP23	496	1	496	0	555.205

GkKUP24	387	13	385	########	371.767
GkKUP25	141	40	140	1.57E-38	137.7
GkKUP26	295	1	293	7.99E-78	252.741
GkKUP27	330	40	313	2.47E-82	266.223
GkKUP28	734	40	732	0	741.174
GkKUP29	92	1	63	4.88E-12	59.4406
GkKUP30	126	27	126	3.60E-31	115.995
GrKUP01	826	31	826	0	1156.14
GrKUP02	249	1	247	########	417.991
GrKUP03	852	1	632	0	1045.48
GrKUP04	485	134	431	########	461.519
GrKUP05	672	53	670	0	610.591
GrKUP06	773	34	771	0	824.377
GrKUP07	738	1	738	0	1238.93
GrKUP08	284	3	61	1.92E-21	93.7628
GrKUP09	743	28	743	0	994.718
GrKUP10	400	104	400	########	446.111
GrKUP11	829	42	829	0	925.767
GrKUP12	496	1	496	0	563.294
GrKUP13	832	13	832	0	897.314
GrKUP14	1012	119	945	0	1517.35
GrKUP15	771	19	769	0	822.836

GrKUP16	399	35	381	########	352.976
GrKUP17	255	2	111	6.08E-33	126.093
GrKUP18	780	1	780	0	1395.74
GrKUP19	561	1	553	0	709.973
GrKUP20	730	58	625	0	674.604
GrKUP21	816	42	814	0	867.134
GrKUP22	887	39	809	0	859.045
GrKUP23	779	16	779	0	910.025
GrKUP24	421	225	347	2.83E-67	228.858
GrKUP25	632	8	580	0	1074.1
GrKUP26	199	5	194	########	326.038
GrKUP27	610	10	610	0	1009.38
GrKUP28	824	19	824	0	1309.73
GrKUP29	251	35	208	4.39E-57	194.575
GrKUP30	303	128	250	4.21E-51	179.938
GrKUP31	195	1	180	########	332.175
GrKUP32	563	11	563	0	993.948
GrKUP33	844	1	829	0	1363.38
GrKUP34	854	1	854	0	1127.64
GrKUP35	671	81	641	########	472.304
GtKUP01	250	134	244	2.20E-48	170.308
GtKUP02	730	5	730	0	941.175

GtKUP03	729	39	727	0	706.121
GtKUP04	82	36	76	8.40E-17	72.8524
GtKUP05	816	39	814	0	861.741
GtKUP06	713	58	608	0	646.1
GtKUP07	225	1	225	########	348.655
GtKUP08	207	1	194	########	385.359
GtKUP09	480	1	480	0	940.046
GtKUP10	205	1	204	2.30E-53	181.947
GtKUP11	407	9	407	0	556.746
GtKUP12	1049	1	737	0	1272.45
GtKUP13	773	34	771	0	828.229
GtKUP14	703	7	657	0	765.826
GtKUP15	186	137	186	7.28E-19	83.2528
GtKUP16	777	13	777	0	1120.68
GtKUP17	766	19	764	0	813.206
GtKUP18	750	17	750	0	1389.08
GtKUP19	795	15	795	0	948.545
GtKUP20	791	22	791	0	809.437
GtKUP21	199	1	199	7.71E-85	265.972
GtKUP22	264	2	263	1.31E-90	285.483
GtKUP23	56	2	55	1.21E-17	74.008
GtKUP24	498	9	496	########	509.669

GtKUP25	774	1	774	0	1111.85
GtKUP26	527	1	524	0	948.494
GtKUP27	308	140	262	3.96E-69	230.014
GtKUP28	856	19	856	0	1566.27
GtKUP29	446	1	444	0	809.078
GtKUP30	249	1	240	########	366.76
GtKUP31	842	1	842	0	1546.63
GtKUP32	755	28	755	0	1072.53
GtKUP33	746	5	746	0	1350.67
HuKUP01	859	22	858	0	1649.47
HuKUP02	763	29	762	0	1061.74
HuKUP03	778	20	775	0	778.153
HuKUP04	781	33	778	0	816.288
HuKUP05	617	3	614	0	564.752
HuKUP06	811	32	808	0	866.749
HuKUP07	741	3	740	0	945.412
HuKUP08	776	2	772	0	828.312
HuKUP09	843	1	842	0	1589
HuKUP10	728	54	621	0	675.76
HuKUP11	779	1	778	0	1535.95
HuKUP12	746	1	745	0	978.54
HuKUP13	794	1	793	0	1168.85

HuKUP14	785	1	784	0	1578.68
HuKUP15	792	1	791	0	1498.2
HuKUP16	777	13	776	0	1172.3
HuKUP17	796	13	795	0	945.079
OsKUP01	809	4	808	0	1238.93
OsKUP02	771	35	768	0	824.762
OsKUP03	749	80	646	0	699.642
OsKUP04	784	1	783	0	1551.72
OsKUP05	743	47	742	0	879.11
OsKUP06	748	48	747	0	865.243
OsKUP07	772	1	771	0	1245.89
OsKUP08	794	1	791	0	1117.62
OsKUP09	812	33	809	0	743.1
OsKUP10	812	40	809	0	719.603
OsKUP11	800	26	797	0	739.633
OsKUP12	174	103	152	1.13E-15	73.3472
OsKUP13	802	58	801	0	877.184
OsKUP14	868	87	867	0	1412.96
OsKUP15	792	10	791	0	911.951
OsKUP16	773	1	772	0	1177.33
OsKUP17	844	18	843	0	1272.86
OsKUP18	779	13	778	0	906.122

OsKUP19	860	21	859	0	1433.76
OsKUP20	812	41	811	0	1411.89
OsKUP21	789	1	786	0	1142.28
OsKUP22	583	1	582	0	675.439
OsKUP23	698	32	602	0	647.64
OsKUP24	740	78	644	0	663.048
OsKUP25	878	29	877	0	1580.52
OsKUP26	716	45	612	0	663.819
OsKUP27	794	1	793	0	919.27
TcKUP01	252	1	230	7.78E-68	223.215
TcKUP02	606	41	553	########	532.01
TcKUP03	781	9	778	0	838.629
TcKUP04	801	104	798	0	715.751
TcKUP05	796	13	795	0	951.627
TcKUP06	861	22	860	0	1642.16
TcKUP07	811	32	808	0	897.18
TcKUP08	843	1	842	0	1592.85
TcKUP09	779	1	778	0	1539.8
TcKUP10	728	54	621	0	683.849
TcKUP11	448	2	447	########	510.522
TcKUP12	777	13	776	0	1155.35
TcKUP13	792	1	791	0	1495.5

TcKUP14	786	1	785	0	1585.61
TcKUP15	746	1	745	0	988.94
TcKUP16	741	3	740	0	934.242
TcKUP17	763	29	762	0	1054.42
TcKUP18	794	1	793	0	1169.24
VvKUP01	747	1	746	0	978.54
VvKUP02	791	13	790	0	947.39
VvKUP03	816	14	813	0	927.225
VvKUP04	686	18	585	0	679.997
VvKUP05	834	13	833	0	1614.42
VvKUP06	780	2	779	0	1565.61
VvKUP07	599	2	598	0	813.289
VvKUP08	161	11	115	6.93E-33	122.241
VvKUP09	418	73	179	6.50E-22	97.9734
VvKUP09	418	1	56	7.51E-18	85.564
VvKUP10	654	5	643	0	934.627
VvKUP11	193	1	126	1.28E-43	154.213
VvKUP12	151	5	130	4.20E-52	176.471
VvKUP13	771	2	768	0	845.563
VvKUP14	194	28	127	7.21E-31	117.921
VvKUP15	774	28	771	0	848.259
VvKUP16	774	26	771	0	840.555

VvKUP17	774	26	771	0	846.718
VvKUP18	179	114	165	8.61E-11	59.4055
VvKUP19	607	25	606	0	1218.05
VvKUP20	289	1	261	########	440.333
VvKUP21	794	1	793	0	1159.22
VvKUP22	157	41	108	3.54E-25	100.202
VvKUP23	69	36	63	2.65E-09	51.0056
VvKUP24	813	1	812	0	1533.25
VvKUP25	784	1	783	0	1606.41
VvKUP26	781	1	769	0	847.187
VvKUP27	127	69	126	1.27E-19	82.9378
VvKUP28	109	11	107	7.63E-28	105.279
VvKUP29	79	19	73	2.56E-15	68.3396
VvKUP30	624	1	623	0	782.473
VvKUP31	240	124	203	2.47E-18	83.3624

Table S6 Sequences and conserved motif annotation of the cotton KUP family.

MEME name	Sequence	Annotation
MEME-1	LAAVVASQAIISGTFSIIKQSLALG<br>CFPRVKVVHTSSKIHGQIYIPEINW	KUP domain
MEME-2	TIMYIWHYGTLKKYEFELHNKVSMDWL<br>LELGPSLGIVRVPGIGLIYTELVSGIPAI	KUP domain
MEME-3	IGJYNIIKWBPHVLKALSPYYI<br>YKFFKRTGKDGWISLGGILLC	KUP domain
MEME-4	LILAYQSLGVVYGDLSTSPLYVYKSTF<br>AGDJQHSETBEEIFGVLSFVFWTLTL	KUP domain
MEME-5	HFVTNLPAFHSVLVFVCIKSVPVPKVPP<br>EERFLFGRVGPKEYRMFRCIVRYGYKDVR	KUP domain
MEME-6	MILCJAVTIGFRDTNRIGNAYGJAV<br>VTVMLVTTCLMTLVMILIWKTNJLLAJ	KUP domain
MEME-7	ABDNGEGGTFALYSLJCRHAKVSLLPNQQA	KUP domain
MEME-8	VLSAVGGLKVAVPALHEDVVVVISVVILVGL	-
MEME-9	SIQJAFTFVVYPCLJLAYMGQAAYLSKHP	KUP domain
MEME-10	RASKJKSFLEKHKSLKTALL<br>VLVLLGTSMVIGDGVLTPAIS	KUP domain
MEME-11	VAYJLGHSYVRAKKGSSFLKKJVIBYGYNFL	KUP domain
MEME-12	LQHFGTHRVGFLFAPIVJLWL	KUP domain
MEME-13	FGSIELJYFSAVLYKFTEGGW	KUP domain

MEME-14	QDSFYKSIPESIFWPVFVIAT	KUP domain
MEME-15	ITGSEAMFADLGHFS	KUP domain
MEME-16	NCREPEVALNVPHASLLEVGM	KUP domain
MEME-17	EFEQQLVESJAEFIRSEAQEA	-
MEME-18	PEDVJGALSLIFYTLTLJPLVKYVFVVLK	KUP domain
MEME-19	KDTGWWRTLRLAFQSJGVVYGDJGTSPL	KUP domain
MEME-20	VFSAVSGLELSMSKEHHQYVEVPIACFIL	KUP domain

-represents no annotation were found.

Table S7 Numbers of exons and introns in the cotton KUP family.

Name	Subfamily	Exon	Intron
GrKUP01	2B	10	9
GrKUP02	5	3	2
GrKUP03	5	12	11
GrKUP04	5	3	2
GrKUP05	1	6	5
GrKUP06	1	8	7
GrKUP07	2A	9	8
GrKUP08	2B	7	6
GrKUP09	2C	9	8
GrKUP10	5	6	5
GrKUP11	2C	11	10
GrKUP12	2B	3	2
GrKUP13	3	9	8
GrKUP14	5	16	15
GrKUP15	1	6	5
GrKUP16	2C	8	7
GrKUP17	2C	4	3
GrKUP18	2B	9	8
GrKUP19	5	11	10
GrKUP20	4	8	7

GrKUP21	1	9	8
GrKUP22	1	11	10
GrKUP23	3	8	7
GrKUP24	5	5	4
GrKUP25	2B	6	5
GrKUP26	2B	3	2
GrKUP27	2B	11	10
GrKUP28	5	13	12
GrKUP29	5	2	1
GrKUP30	5	3	2
GrKUP31	2A	3	2
GrKUP32	2A	6	5
GrKUP33	2B	8	7
GrKUP34	2B	10	9
GrKUP35	1	9	8
GaKUP01	2C	10	9
GaKUP02	2B	8	7
GaKUP03	2A	9	8
GaKUP04	2A	3	2
GaKUP05	1	9	8
GaKUP06	3	9	8
GaKUP07	2B	8	7

GaKUP08	5	10	9
GaKUP09	2A	9	8
GaKUP10	2B	7	6
GaKUP11	2B	9	8
GaKUP12	1	12	11
GaKUP13	2B	10	9
GaKUP14	4	8	7
GaKUP15	5	10	9
GaKUP16	2C	9	8
GaKUP17	2B	3	2
GaKUP18	2B	4	3
GaKUP19	2C	9	8
GaKUP20	1	8	7
GaKUP21	1	11	10
GaKUP22	2A	15	14
GaKUP23	2B	8	7
GaKUP24	3	8	7
GaKUP25	5	10	9
GaKUP26	1	6	5
GbKUP01	2C	12	11
GbKUP02	2B	7	6
GbKUP03	4	8	7

GbKUP04	1	9	8
GbKUP05	1	11	10
GbKUP06	2A	13	12
GbKUP07	3	7	6
GbKUP08	5	10	9
GbKUP09	2B	8	7
GbKUP10	2B	9	8
GbKUP11	5	9	8
GbKUP12	2A	9	8
GbKUP13	2B	7	6
GbKUP14	2B	9	8
GbKUP15	1	9	8
GbKUP16	2C	9	8
GbKUP17	2B	8	7
GbKUP18	2C	8	7
GbKUP19	1	5	4
GbKUP20	1	6	5
GbKUP21	1	6	5
GbKUP22	5	10	9
GbKUP23	3	13	12
GbKUP24	2B	8	7
GbKUP25	2C	8	7

GbKUP26	2B	8	7
GbKUP27	5	10	9
GbKUP28	4	8	7
GbKUP29	1	9	8
GbKUP30	5	2	1
GbKUP31	3	8	7
GbKUP32	2A	9	8
GbKUP33	1	8	7
GbKUP34	2B	9	8
GbKUP35	2B	7	6
GbKUP36	2A	9	8
GbKUP37	5	1	0
GbKUP38	5	10	9
GbKUP39	2B	10	9
GbKUP40	5	9	8
GbKUP41	5	2	1
GbKUP42	2C	9	8
GbKUP43	2B	8	7
GbKUP44	2C	8	7
GbKUP45	5	2	1
GbKUP46	1	8	7
GbKUP47	1	7	6

GbKUP48	1	6	5
GbKUP49	5	10	9
GbKUP50	3	8	7
GbKUP51	2B	7	6
GhKUP01	2C	11	10
GhKUP02	2B	7	6
GhKUP03	4	8	7
GhKUP04	1	9	8
GhKUP05	1	11	10
GhKUP06	2A	9	8
GhKUP07	3	8	7
GhKUP08	5	10	9
GhKUP09	2B	8	7
GhKUP10	2B	9	8
GhKUP11	5	9	8
GhKUP12	2A	9	8
GhKUP13	2B	7	6
GhKUP14	2B	9	8
GhKUP15	1	9	8
GhKUP16	2C	9	8
GhKUP17	2B	8	7
GhKUP18	2C	9	8

GhKUP19	2A	10	9
GhKUP20	1	6	5
GhKUP21	1	6	5
GhKUP22	1	6	5
GhKUP23	5	10	9
GhKUP24	3	8	7
GhKUP25	2B	8	7
GhKUP26	2C	7	6
GhKUP27	2B	8	7
GhKUP28	5	8	7
GhKUP29	4	8	7
GhKUP30	1	9	8
GhKUP31	5	2	1
GhKUP32	3	10	9
GhKUP33	2A	9	8
GhKUP34	1	8	7
GhKUP35	2B	9	8
GhKUP36	2B	7	6
GhKUP37	2A	9	8
GhKUP38	5	1	0
GhKUP39	5	10	9
GhKUP40	2B	10	9

GhKUP41	5	9	8
GhKUP42	5	2	1
GhKUP43	5	2	1
GhKUP44	2C	9	8
GhKUP45	2B	8	7
GhKUP46	2C	8	7
GhKUP47	5	2	1
GhKUP48	1	8	7
GhKUP49	1	7	6
GhKUP50	1	6	5
GhKUP51	5	10	9
GhKUP52	3	8	7
GhKUP53	2B	5	4

Table S8 Numbers of stress-responsive regulatory elements in the promoter regions of *GbKUP*s and *GhKUP*s.

Name	Subfamily	ABRE	DSR	GARE	LTR	MBS
GbKUP05	1	1	0	0	0	1
GbKUP19	1	0	0	1	0	0
GbKUP20	1	1	0	0	0	0
GbKUP21	1	0	0	1	0	1
GbKUP46	1	0	0	2	0	0
GbKUP47	1	1	0	0	0	1
GhKUP04	1	1	0	0	0	0
GhKUP05	1	1	0	0	0	1
GhKUP21	1	1	0	0	0	0
GhKUP22	1	0	0	0	1	1
GhKUP48	1	0	0	2	0	0
GhKUP49	1	1	0	0	0	1
GhKUP50	1	1	0	0	0	0
GbKUP06	2A	0	0	0	1	0
GbKUP32	2A	0	0	0	1	0
GbKUP36	2A	0	0	1	0	0
GhKUP06	2A	0	0	0	1	0
GhKUP19	2A	1	1	1	0	0
GhKUP33	2A	0	0	0	1	0

GhKUP37	2A	0	0	1	0	0
GbKUP09	2B	0	0	0	0	1
GbKUP10	2B	0	1	0	0	1
GbKUP13	2B	0	1	1	0	0
GbKUP14	2B	1	0	0	0	0
GbKUP17	2B	0	1	0	0	0
GbKUP24	2B	2	1	0	0	0
GbKUP26	2B	0	1	0	0	1
GbKUP34	2B	1	0	0	0	0
GbKUP35	2B	0	1	2	0	0
GbKUP39	2B	1	1	0	0	1
GbKUP43	2B	0	1	0	0	0
GbKUP51	2B	2	1	0	0	1
GhKUP09	2B	1	0	0	0	1
GhKUP10	2B	0	1	0	0	1
GhKUP13	2B	0	1	1	0	0
GhKUP14	2B	1	0	0	0	0
GhKUP17	2B	0	1	0	0	0
GhKUP25	2B	2	0	0	1	0
GhKUP27	2B	0	1	0	0	1
GhKUP35	2B	1	0	0	0	0
GhKUP36	2B	0	1	2	0	0

GhKUP40	2B	1	1	0	0	1
GhKUP45	2B	0	1	0	0	0
GhKUP53	2B	1	0	2	1	1
GbKUP01	2C	1	1	1	0	0
GbKUP18	2C	1	0	0	0	0
GbKUP25	2C	1	1	1	1	0
GbKUP44	2C	1	1	1	0	0
GhKUP01	2C	1	1	1	0	0
GhKUP26	2C	1	1	1	0	0
GhKUP46	2C	1	0	0	0	0
GbKUP07	3	0	0	0	0	1
GbKUP23	3	0	0	1	0	0
GbKUP31	3	1	0	0	1	0
GbKUP50	3	1	0	2	0	0
GhKUP07	3	0	0	1	0	1
GhKUP24	3	0	0	1	0	0
GhKUP32	3	1	0	0	1	0
GhKUP52	3	1	0	2	0	0
GbKUP03	4	1	0	0	0	0
GhKUP03	4	1	0	0	0	0
GhKUP29	4	0	0	0	0	1
GbKUP08	5	0	1	0	0	0

GbKUP22	5	1	1	0	0	0
GbKUP27	5	1	2	0	1	1
GbKUP30	5	0	0	0	1	1
GbKUP37	5	0	0	1	0	1
GbKUP38	5	1	0	1	0	0
GbKUP40	5	0	0	1	0	0
GbKUP41	5	0	1	1	1	1
GbKUP45	5	0	0	1	0	0
GbKUP49	5	1	1	0	0	0
GhKUP08	5	1	0	0	0	0
GhKUP11	5	0	0	1	0	0
GhKUP23	5	1	1	0	0	0
GhKUP28	5	1	0	0	3	2
GhKUP31	5	0	0	0	0	1
GhKUP38	5	0	0	2	0	1
GhKUP39	5	1	0	0	0	0
GhKUP41	5	0	0	1	0	0
GhKUP42	5	0	0	0	0	1
GhKUP43	5	1	1	1	1	1
GhKUP47	5	0	0	1	0	0
GhKUP51	5	1	1	0	0	0

Table S9 Genomic locations of *KUP* genes in *G. raimondii, G. arboreum, G. barbadense*, and *G. hirsutum*.

Name	Subfamily	Chromosome	Start (bp)	End (bp)
GrKUP01	2B	Gr05	25427153	25431990
GrKUP02	5	Gr05	40327831	40329297
GrKUP03	5	Gr05	40330337	40336291
GrKUP04	5	Gr05	44149820	44151462
GrKUP05	1	Gr11	12539014	12541929
GrKUP06	1	Gr11	55278467	55282018
GrKUP07	2A	Gr11	60684502	60687773
GrKUP08	2B	Gr08	7672162	7674172
GrKUP09	2C	Gr08	56436333	56440139
GrKUP10	5	Gr10	50087004	50088452
GrKUP11	2C	Gr10	58364458	58368848
GrKUP12	2B	Gr12	532523	535313
GrKUP13	3	Gr12	50491897	50496324
GrKUP14	5	Gr12	55617192	55626950
GrKUP15	1	Gr12	56241244	56245417
GrKUP16	2C	Gr01	11971318	11980364
GrKUP17	2C	Gr01	11981003	11981939
GrKUP18	2B	Gr01	34007933	34013835
GrKUP19	5	Gr01	34701041	34704929
GrKUP20	4	Gr01	36906752	36910806

GrKUP21	1	Gr01	50244348	50247574
GrKUP22	1	Gr01	50338325	50342249
GrKUP23	3	Gr03	132031	135135
GrKUP24	5	Gr03	39929223	39933118
GrKUP25	2B	Gr09	7558457	7565221
GrKUP26	2B	Gr09	7566822	7567569
GrKUP27	2B	Gr04	1361961	1365722
GrKUP28	5	Gr04	3927236	3932088
GrKUP29	5	Gr04	21411011	21412164
GrKUP30	5	Gr04	22100761	22101938
GrKUP31	2A	Gr04	37175006	37176489
GrKUP32	2A	Gr04	37177142	37179984
GrKUP33	2B	Gr04	42363088	42367228
GrKUP34	2B	Gr04	48653882	48658508
GrKUP35	1	Gr04	49162768	49167198
GaKUP01	2C	Ga01	17166986	17170428
GaKUP02	2B	Ga01	67717483	67723393
GaKUP03	2A	Ga01	79227940	79232149
GaKUP04	2A	Ga02	8277813	8279500
GaKUP05	1	Ga02	89981191	89985611
GaKUP06	3	Ga03	1.36E+08	1.36E+08
GaKUP07	2B	Ga04	589390	593279

GaKUP08	5	Ga04	2310335	2315137
GaKUP09	2A	Ga04	78549494	78554493
GaKUP10	2B	Ga04	86968955	86973009
GaKUP11	2B	Ga04	96882250	96886888
GaKUP12	1	Ga04	97389760	97392953
GaKUP13	2B	Ga05	31965699	31970537
GaKUP14	4	Ga05	53052682	53056788
GaKUP15	5	Ga05	70490242	70497777
GaKUP16	2C	Ga08	1.23E+08	1.23E+08
GaKUP17	2B	Ga09	8490334	8491943
GaKUP18	2B	Ga09	8515759	8522246
GaKUP19	2C	Ga10	1.25E+08	1.25E+08
GaKUP20	1	Ga11	18328963	18331835
GaKUP21	1	Ga11	1.12E+08	1.12E+08
GaKUP22	2A	Ga11	1.19E+08	1.19E+08
GaKUP23	2B	Ga12	536174	539694
GaKUP24	3	Ga12	97734054	97738458
GaKUP25	5	Ga12	1.01E+08	1.01E+08
GaKUP26	1	Ga12	1.02E+08	1.02E+08
GbKUP01	2C	GbAt01	15546264	15549731
GbKUP02	2B	GbAt01	60809409	60814623
GbKUP03	4	GbAt01	71711423	71715526

GbKUP04	1	GbAt01	1.07E+08	1.07E+08
GbKUP05	1	GbAt01	1.07E+08	1.07E+08
GbKUP06	2A	GbAt02	47665818	47675716
GbKUP07	3	GbAt03	1.08E+08	1.08E+08
GbKUP08	5	GbAt04	77786737	77791421
GbKUP09	2B	GbAt04	80635771	80639891
GbKUP10	2B	GbAt05	31611318	31615914
GbKUP11	5	GbAt05	72025684	72031735
GbKUP12	2A	GbAt05	77332804	77337805
GbKUP13	2B	GbAt05	97116839	97120895
GbKUP14	2B	GbAt05	1.06E+08	1.06E+08
GbKUP15	1	GbAt05	1.06E+08	1.06E+08
GbKUP16	2C	GbAt08	1.15E+08	1.15E+08
GbKUP17	2B	GbAt09	7781228	7801407
GbKUP18	2C	GbAt10	4554070	4557644
GbKUP19	1	GbAt11	12170281	12174038
GbKUP20	1	GbAt11	1E+08	1E+08
GbKUP21	1	GbAt12	1171927	1176187
GbKUP22	5	GbAt12	1893877	1900101
GbKUP23	3	GbAt12	9022907	9037687
GbKUP24	2B	GbAt12	1.01E+08	1.01E+08
GbKUP25	2C	GbDt01	12883374	12886232

GbKUP26	2B	GbDt01	39804301	39810207
GbKUP27	5	GbDt01	40519753	40545481
GbKUP28	4	GbDt01	42119665	42123619
GbKUP29	1	GbDt01	56851785	56854991
GbKUP30	5	GbDt02	24535115	24535993
GbKUP31	3	GbDt02	68765461	68767821
GbKUP32	2A	GbDt03	31993065	31997501
GbKUP33	1	GbDt04	1052795	1055771
GbKUP34	2B	GbDt04	1564742	1569382
GbKUP35	2B	GbDt04	8679566	8683618
GbKUP36	2A	GbDt04	13889644	13894616
GbKUP37	5	GbDt04	35106345	35106809
GbKUP38	5	GbDt04	53254918	53259776
GbKUP39	2B	GbDt05	27122199	27127038
GbKUP40	5	GbDt05	46031639	46037652
GbKUP41	5	GbDt08	50541744	50542475
GbKUP42	2C	GbDt08	62232261	62236070
GbKUP43	2B	GbDt09	9027009	9034691
GbKUP44	2C	GbDt10	4349149	4352760
GbKUP45	5	GbDt10	12724931	12725875
GbKUP46	1	GbDt11	11012328	11015993
GbKUP47	1	GbDt11	58695881	58698799

GbKUP48	1	GbDt12	1135668	1139922
GbKUP49	5	GbDt12	1752144	1758348
GbKUP50	3	GbDt12	6859924	6864358
GbKUP51	2B	GbDt12	60247752	60251201
GhKUP01	2C	GhAt01	15754817	15758258
GhKUP02	2B	GhAt01	62497043	62502257
GhKUP03	4	GhAt01	73909232	73913335
GhKUP04	1	GhAt01	1.09E+08	1.09E+08
GhKUP05	1	GhAt01	1.09E+08	1.09E+08
GhKUP06	2A	GhAt02	47769561	47773761
GhKUP07	3	GhAt03	1.11E+08	1.11E+08
GhKUP08	5	GhAt04	83361507	83366309
GhKUP09	2B	GhAt04	86485771	86489660
GhKUP10	2B	GhAt05	31213646	31218243
GhKUP11	5	GhAt05	76179566	76185614
GhKUP12	2A	GhAt05	81761800	81766801
GhKUP13	2B	GhAt05	99645641	99649697
GhKUP14	2B	GhAt05	1.09E+08	1.09E+08
GhKUP15	1	GhAt05	1.1E+08	1.1E+08
GhKUP16	2C	GhAt08	1.19E+08	1.19E+08
GhKUP17	2B	GhAt09	7694470	7720815
GhKUP18	2C	GhAt10	4511862	4515874

GhKUP19	2A	GhAt11	5782805	5786174
GhKUP20	1	GhAt11	12151308	12155077
GhKUP21	1	GhAt11	1.06E+08	1.06E+08
GhKUP22	1	GhAt12	1216243	1220419
GhKUP23	5	GhAt12	1954167	1960370
GhKUP24	3	GhAt12	8911937	8916342
GhKUP25	2B	GhAt12	1.07E+08	1.07E+08
GhKUP26	2C	GhDt01	12668493	12671351
GhKUP27	2B	GhDt01	40173797	40179699
GhKUP28	5	GhDt01	41116580	41120687
GhKUP29	4	GhDt01	42780156	42784156
GhKUP30	1	GhDt01	57884167	57887394
GhKUP31	5	GhDt02	25148294	25149172
GhKUP32	3	GhDt02	69645995	69657607
GhKUP33	2A	GhDt03	32048052	32052254
GhKUP34	1	GhDt04	1215747	1218714
GhKUP35	2B	GhDt04	1721053	1725693
GhKUP36	2B	GhDt04	9020698	9024750
GhKUP37	2A	GhDt04	14168883	14173855
GhKUP38	5	GhDt04	34707753	34708145
GhKUP39	5	GhDt04	52812556	52817413
GhKUP40	2B	GhDt05	26827666	26832505

GhKUP41	5	GhDt05	45234176	45240189
GhKUP42	5	GhDt05	49498508	49499494
GhKUP43	5	GhDt08	52024172	52024903
GhKUP44	2C	GhDt08	63658750	63662559
GhKUP45	2B	GhDt09	7528012	7535402
GhKUP46	2C	GhDt10	4323238	4326846
GhKUP47	5	GhDt10	12508515	12509459
GhKUP48	1	GhDt11	11060682	11064308
GhKUP49	1	GhDt11	58083183	58086101
GhKUP50	1	GhDt12	1160747	1165004
GhKUP51	5	GhDt12	1827670	1833874
GhKUP52	3	GhDt12	6989350	6993783
GhKUP53	2B	GhDt12	61183815	61186600

Table S10 Ka/Ks analysis and estimated divergence time of the duplicated *KUPs* in *G. raimondii*, *G. arboreum*, *G. barbadense*, and *G. hirsutum*.

Species	Duplicated gene 1	Duplicated gene 2	Subfamily	Ka	Ks	Ka/Ks	Purifying selection	Duplicated type	Age(MYA)
G.raimondii	GrKUP23	GrKUP13	3	0.105921	0.48411	0.218796	Yes	Segmental	93.098
	GrKUP34	GrKUP01	2B	0.048023	0.352761	0.136136	Yes	Segmental	67.83864
	GrKUP33	GrKUP25	2B	0.097277	0.609627	0.159568	Yes	Segmental	117.236
	GrKUP28	GrKUP14	5	0.14262	0.529252	0.269475	Yes	Segmental	101.7792
	GrKUP06	GrKUP15	1	0.219734	1.608866	0.136577	Yes	Segmental	309.3973
G.hirsutum	GhKUP28	GhKUP41	5	0.157072	0.576886	0.272275	Yes	Segmental	110.9396
	GhKUP32	GhKUP52	3	0.105456	0.475768	0.221655	Yes	Segmental	91.49378
	GhKUP35	GhKUP40	2B	0.047455	0.354805	0.133749	Yes	Segmental	68.23167
	GhKUP36	GhKUP45	2B	0.067352	0.547884	0.12293	Yes	Segmental	105.3624
	GhKUP39	GhKUP51	5	0.079354	0.466552	0.170087	Yes	Segmental	89.7215
	GhKUP07	GhKUP24	3	0.107362	0.368495	0.291352	Yes	Segmental	70.86438

	GhKUP08	GhKUP23	5	0.07668	0.464798	0.164975	Yes	Segmental	89.38422
	GhKUP10	GhKUP14	2B	0.04858	0.373407	0.1301	Yes	Segmental	71.80897
	GhKUP13	GhKUP17	2B	0.072126	0.551612	0.130754	Yes	Segmental	106.0791
G.barbadense	GbKUP27	GbKUP40	5	0.148204	0.571743	0.259214	Yes	Segmental	109.9507
	GbKUP31	GbKUP50	3	0.101018	0.401903	0.251349	Yes	Segmental	77.28901
	GbKUP34	GbKUP39	2B	0.048624	0.341636	0.142328	Yes	Segmental	65.69915
	GbKUP35	GbKUP43	2B	0.068286	0.546233	0.125012	Yes	Segmental	105.0449
	GbKUP38	GbKUP49	5	0.07711	0.462638	0.166674	Yes	Segmental	88.96879
	GbKUP07	GbKUP23	3	0.098479	0.322901	0.304983	Yes	Segmental	62.09637
	GbKUP08	GbKUP22	5	0.0702	0.451548	0.155465	Yes	Segmental	86.83609
	GbKUP10	GbKUP14	2B	0.049174	0.370574	0.132698	Yes	Segmental	71.26419
	GbKUP13	GbKUP17	2B	0.071824	0.564541	0.127226	Yes	Segmental	108.5656
G.arboreum	GaKUP06	GaKUP24	3	0.112298	0.475849	0.235994	Yes	Segmental	91.50934

GaKUP11	GaKUP13	2B	0.048302	0.365802	0.132044	Yes	Segmental	70.34654
GaKUP10	GaKUP17	2B	0.083568	0.631433	0.132347	Yes	Segmental	121.4294

Table S11 Orthologous gene groups of the KUP members in *G. barbadense*, *G. hirsutum*, *G. arboretum*, and *G. raimondii*.

Genes of Ga genome	Genes of GbAt subgenome	Genes of GbDt subgenome	Genes of GhAt subgenome	Genes of GhDt subgenome	Genes of Gr genome	Subfamily
GaKUP01	GbKUP01	GbKUP25	GhKUP01	GhKUP26	GrKUP16	2C
GaKUP02	GbKUP02	GbKUP26	GhKUP02	GhKUP27	GrKUP18	2B
-	GbKUP03	GbKUP28	GhKUP03	GhKUP29	GrKUP20	4
GaKUP05	GbKUP04	GbKUP29	GhKUP04	GhKUP30	GrKUP22	1
-	GbKUP05	-	GhKUP05	-	-	1
GaKUP03	GbKUP06	GbKUP32	GhKUP06	GhKUP33	-	2A
GaKUP06	GbKUP07	GbKUP31	GhKUP07	GhKUP32	GrKUP23	3
GaKUP08	GbKUP08	GbKUP38	GhKUP08	GhKUP39	GrKUP28	5
GaKUP07	GbKUP09	-	GhKUP09	-	GrKUP27	2B
GaKUP13	GbKUP10	GbKUP39	GhKUP10	GhKUP40	GrKUP01	2B
GaKUP15	GbKUP11	GbKUP40	GhKUP11	GhKUP41	GrKUP03	5

GaKUP09	GbKUP12	GbKUP36	GhKUP12	GhKUP37	GrKUP32	2A
GaKUP10	GbKUP13	GbKUP35	GhKUP13	GhKUP36	GrKUP33	2B
GaKUP11	GbKUP14	GbKUP34	GhKUP14	GhKUP35	GrKUP34	2B
GaKUP12	GbKUP15	GbKUP33	GhKUP15	GhKUP34	GrKUP35	1
GaKUP16	GbKUP16	GbKUP42	GhKUP16	GhKUP44	GrKUP09	2C
GaKUP17	GbKUP17	GbKUP43	GhKUP17	GhKUP45	GrKUP25	2B
GaKUP19	GbKUP18	GbKUP44	GhKUP18	GhKUP46	GrKUP11	2C
GaKUP21	GbKUP19	GbKUP46	GhKUP20	GhKUP48	GrKUP06	1
GaKUP20	GbKUP20	GbKUP47	GhKUP21	GhKUP49	GrKUP05	1
GaKUP26	GbKUP21	GbKUP48	GhKUP22	GhKUP50	GrKUP15	1
GaKUP25	GbKUP22	GbKUP49	GhKUP23	GhKUP51	GrKUP14	5
GaKUP24	GbKUP23	GbKUP50	GhKUP24	GhKUP52	GrKUP13	3
GaKUP23	GbKUP24	GbKUP51	GhKUP25	GhKUP53	GrKUP12	2B

GaKUP22	-	-	GhKUP19	-	GrKUP07	2A
-	-	GbKUP27	-	GhKUP28	GrKUP19	5
-	-	GbKUP30	-	GhKUP31	GrKUP24	5
-	-	GbKUP37	-	GhKUP38	-	5
-	-	-	-	GhKUP42	GrKUP04	5
-	-	GbKUP41	-	GhKUP43	-	5
-	-	GbKUP45	-	GhKUP47	-	5

Table S12 Ka/Ks analysis for the KUP orthologous gene pairs in *G. raimondii*, *G. arboreum*, *G. barbadense*, and *G. hirsutum*.

Gene 1	Gene 2	Subfamily	Ka	Ks	Ka/Ks
GaKUP01	GrKUP16	2C	0.155742	0.202484	0.769158
GaKUP02	GrKUP18	2B	0.003009	0.034694	0.086727
GaKUP05	GrKUP22	1	0.037	0.073754	0.501672
GaKUP06	GrKUP23	3	0.019635	0.051459	0.381572
GaKUP07	GrKUP27	2B	0.04394	0.089457	0.491187
GaKUP08	GrKUP28	5	0.063708	0.066969	0.951312
GaKUP09	GrKUP32	2A	0.015144	0.049548	0.305635
GaKUP10	GrKUP33	2B	0.020927	0.03286	0.636866
GaKUP11	GrKUP34	2B	0.003908	0.022759	0.171693
GaKUP12	GrKUP35	1	0.257727	0.345403	0.746161
GaKUP13	GrKUP01	2B	0.008761	0.046728	0.187486
GaKUP15	GrKUP03	5	0.03598	0.077013	0.467191
GaKUP16	GrKUP09	2C	0.025106	0.057049	0.440075
GaKUP17	GrKUP25	2B	0.017437	0.099478	0.175287
GaKUP19	GrKUP11	2C	0.011687	0.050327	0.232216
GaKUP20	GrKUP05	1	0.043636	0.093617	0.466115
GaKUP21	GrKUP06	1	0.026092	0.062599	0.416814
GaKUP22	GrKUP07	2A	0.053976	0.125995	0.428402
GaKUP23	GrKUP12	2B	0.018727	0.065146	0.287468

GaKUP24	GrKUP23	3	0.100086	0.479114	0.208898
GaKUP24	GrKUP13	3	0.016434	0.060373	0.272206
GaKUP25	GrKUP14	5	0.018446	0.047499	0.388341
GaKUP26	GrKUP15	1	0.010497	0.044982	0.233361
GbKUP01	GaKUP01	2C	0.133722	0.17181	0.778309
GbKUP02	GaKUP02	2B	0.001429	0.013539	0.105551
GbKUP06	GaKUP03	2A	0.007289	0.011039	0.660296
GbKUP04	GaKUP05	1	0.012161	0.01633	0.744735
GbKUP07	GaKUP06	3	0.019159	0.019217	0.996944
GbKUP09	GaKUP07	2B	0.003387	0.00539	0.628403
GbKUP08	GaKUP08	5	0.006511	0.012002	0.542483
GbKUP12	GaKUP09	2A	0.00112	0.003471	0.322814
GbKUP13	GaKUP10	2B	0.002828	0.008985	0.314789
GbKUP14	GaKUP11	2B	0.001114	0.003463	0.321639
GbKUP15	GaKUP12	1	0.356899	0.490333	0.72787
GbKUP10	GaKUP13	2B	0.001665	0.013815	0.120533
GbKUP11	GaKUP15	5	0.020191	0.025513	0.791386
GbKUP16	GaKUP16	2C	0.002899	0.003733	0.776795
GbKUP17	GaKUP17	2B	0.007864	0.015997	0.491624
GbKUP18	GaKUP19	2C	0.004537	0.010412	0.43575
GbKUP20	GaKUP20	1	0.017266	0.020177	0.855754
GbKUP19	GaKUP21	1	0.005364	0.0125	0.429111

GbKUP24	GaKUP23	2B	0.003325	0.010702	0.310729
GbKUP23	GaKUP24	3	0.069625	0.088665	0.785255
GbKUP22	GaKUP25	5	0.001527	0	#NAME?
GbKUP21	GaKUP26	1	0.002245	0.009311	0.241075
GbKUP01	GbKUP25	2C	0.097774	0.128833	0.758918
GbKUP02	GbKUP26	2B	0.002861	0.041366	0.069165
GbKUP03	GbKUP28	4	0.007842	0.043248	0.181317
GbKUP04	GbKUP29	1	0.010679	0.048291	0.221146
GbKUP07	GbKUP31	3	0.023606	0.034529	0.683675
GbKUP23	GbKUP31	3	0.118049	0.416976	0.283107
GbKUP06	GbKUP32	2A	0.011314	0.040148	0.281807
GbKUP14	GbKUP34	2B	0.002788	0.021002	0.132762
GbKUP13	GbKUP35	2B	0.006234	0.025469	0.244765
GbKUP12	GbKUP36	2A	0.00393	0.028189	0.13943
GbKUP08	GbKUP38	5	0.012543	0.025865	0.484966
GbKUP10	GbKUP39	2B	0.010049	0.049473	0.203129
GbKUP11	GbKUP40	5	0.008334	0.03436	0.242551
GbKUP16	GbKUP42	2C	0.007557	0.034357	0.219947
GbKUP17	GbKUP43	2B	0.006903	0.051197	0.134831
GbKUP18	GbKUP44	2C	0.004841	0.048529	0.099748
GbKUP19	GbKUP46	1	0.02725	0.070497	0.386535
GbKUP20	GbKUP47	1	0.025978	0.065195	0.398473

GbKUP21	GbKUP48	1	0.01107	0.05289	0.20931
GbKUP22	GbKUP49	5	0.003569	0.025082	0.142297
GbKUP23	GbKUP50	3	0.06502	0.11186	0.581263
GbKUP24	GbKUP51	2B	0.019319	0.078556	0.245925
GbKUP39	GrKUP01	2B	0.001606	0.004974	0.322922
GbKUP40	GrKUP03	5	0.02818	0.05308	0.53089
GbKUP47	GrKUP05	1	0.031331	0.046857	0.66865
GbKUP46	GrKUP06	1	0.001699	0.007288	0.233148
GbKUP42	GrKUP09	2C	0.023571	0.037964	0.620876
GbKUP44	GrKUP11	2C	0.006677	0.003746	1.782153
GbKUP51	GrKUP12	2B	0.005013	0.013133	0.381707
GbKUP50	GrKUP13	3	0.008022	0.020737	0.386862
GbKUP49	GrKUP14	5	0.016306	0.026135	0.623941
GbKUP48	GrKUP15	1	0.004523	0.016884	0.267917
GbKUP25	GrKUP16	2C	0.12812	0.135467	0.945765
GbKUP26	GrKUP18	2B	6.01E-04	0.003774	0.159254
GbKUP27	GrKUP19	5	0.145246	0.187188	0.775936
GbKUP28	GrKUP20	4	0.004816	0.00383	1.257615
GbKUP29	GrKUP22	1	0.019466	0.041358	0.470663
GbKUP31	GrKUP23	3	0.020072	0.04268	0.470296
GbKUP50	GrKUP23	3	0.097186	0.476443	0.203982
GbKUP30	GrKUP24	5	0.018682	0.017665	1.057558

GbKUP43	GrKUP25	2B	0.010422	0.027442	0.379785
GbKUP38	GrKUP28	5	0.057238	0.05072	1.128495
GbKUP36	GrKUP32	2A	0.014336	0.023269	0.616077
GbKUP35	GrKUP33	2B	0.019721	0.021554	0.914958
GbKUP34	GrKUP34	2B	0.001115	0.005187	0.214914
GbKUP33	GrKUP35	1	0.224028	0.289066	0.775006
GhKUP01	GaKUP01	2C	0.138936	0.182185	0.762607
GhKUP02	GaKUP02	2B	0.001429	0.013539	0.105551
GhKUP06	GaKUP03	2A	0.003421	0.003516	0.973011
GhKUP04	GaKUP05	1	0.012161	0.01633	0.744735
GhKUP07	GaKUP06	3	0.030299	0.031805	0.95265
GhKUP09	GaKUP07	2B	0.002823	0.005381	0.524606
GhKUP08	GaKUP08	5	0.003079	0.006529	0.471602
GhKUP12	GaKUP09	2A	0.001681	0.003472	0.484216
GhKUP13	GaKUP10	2B	0.001696	0.007181	0.236104
GhKUP14	GaKUP11	2B	0.001114	0.003463	0.321639
GhKUP15	GaKUP12	1	0.364405	0.502964	0.724515
GhKUP10	GaKUP13	2B	0.00111	0.008605	0.128967
GhKUP11	GaKUP15	5	0.020772	0.023747	0.874722
GhKUP16	GaKUP16	2C	0.003481	0.003733	0.932514
GhKUP17	GaKUP17	2B	0.007864	0.015997	0.491624
GhKUP18	GaKUP19	2C	0.005146	0.012303	0.418295

GhKUP21	GaKUP20	1	0.017998	0.017683	1.017801
GhKUP20	GaKUP21	1	0.02269	0.03351	0.677109
GhKUP19	GaKUP22	2A	0.025712	0.049966	0.51459
GhKUP25	GaKUP23	2B	0.00277	0.0107	0.258895
GhKUP24	GaKUP24	3	0.007129	0.016339	0.436295
GhKUP23	GaKUP25	5	0.001527	0.001647	0.927699
GhKUP22	GaKUP26	1	0.002244	0.005578	0.402305
GhKUP01	GhKUP26	2C	0.099686	0.137384	0.7256
GhKUP02	GhKUP27	2B	0.004657	0.042483	0.10962
GhKUP03	GhKUP29	4	0.00784	0.043277	0.181162
GhKUP04	GhKUP30	1	0.012219	0.053422	0.228718
GhKUP07	GhKUP32	3	0.027274	0.037244	0.7323
GhKUP06	GhKUP33	2A	0.007431	0.035946	0.206721
GhKUP14	GhKUP35	2B	0.003348	0.024538	0.136441
GhKUP13	GhKUP36	2B	0.003961	0.025476	0.155462
GhKUP12	GhKUP37	2A	0.003929	0.030022	0.130869
GhKUP08	GhKUP39	5	0.011362	0.026387	0.430602
GhKUP10	GhKUP40	2B	0.008927	0.051287	0.17406
GhKUP11	GhKUP41	5	0.008867	0.032661	0.2715
GhKUP16	GhKUP44	2C	0.006973	0.034357	0.202949
GhKUP17	GhKUP45	2B	0.006325	0.047297	0.133721
GhKUP18	GhKUP46	2C	0.007883	0.054543	0.144533

GhKUP20	GhKUP48	1	0.043377	0.084854	0.511196
GhKUP21	GhKUP49	1	0.027634	0.059578	0.463822
GhKUP22	GhKUP50	1	0.009922	0.052992	0.187232
GhKUP23	GhKUP51	5	0.003569	0.026785	0.133253
GhKUP24	GhKUP52	3	0.003311	0.034153	0.096958
GhKUP25	GhKUP53	2B	0.02253	0.075198	0.299609
GhKUP40	GrKUP01	2B	0.00107	0.011654	0.091855
GhKUP41	GrKUP03	5	0.02818	0.047839	0.589051
GhKUP42	GrKUP04	5	0.045939	0.072439	0.63417
GhKUP49	GrKUP05	1	0.031331	0.042474	0.737646
GhKUP48	GrKUP06	1	0.001699	0.007288	0.233148
GhKUP44	GrKUP09	2C	0.02236	0.037964	0.588966
GhKUP46	GrKUP11	2C	0.008506	0.013208	0.643976
GhKUP53	GrKUP12	2B	0.0062	0.02293	0.270379
GhKUP52	GrKUP13	3	0.007464	0.018919	0.394528
GhKUP51	GrKUP14	5	0.016305	0.026142	0.623707
GhKUP50	GrKUP15	1	0.004522	0.013111	0.344911
GhKUP26	GrKUP16	2C	0.12812	0.135467	0.945765
GhKUP27	GrKUP18	2B	0.002106	0.004714	0.446819
GhKUP29	GrKUP20	4	0.004815	0.003832	1.25656
GhKUP30	GrKUP22	1	0.02094	0.046481	0.450494
GhKUP32	GrKUP23	3	0.006646	0.015412	0.431185

GhKUP52	GrKUP23	3	0.097196	0.472861	0.205548
GhKUP31	GrKUP24	5	0.016969	0.017648	0.961535
GhKUP45	GrKUP25	2B	0.010422	0.027442	0.379785
GhKUP39	GrKUP28	5	0.060875	0.06674	0.91212
GhKUP37	GrKUP32	2A	0.013519	0.025885	0.522277
GhKUP36	GrKUP33	2B	0.019752	0.023445	0.842447
GhKUP35	GrKUP34	2B	0.001673	0.008657	0.193268
GhKUP34	GrKUP35	1	0.190896	0.217385	0.878147
