# Supplementary material for: Molecular Evolution and Expansion of the KUP Family in the Allopolyploid Cotton Species Gossypium hirsutum and Gossypium barbadense
Source: Front Plant Sci. 2020 Sep 30;11:545042. doi: 10.3389/fpls.2020.545042 (PMC7554350; doi:10.3389/fpls.2020.545042)
Supplement: Supplementary file 2 [file DataSheet_2.pdf]

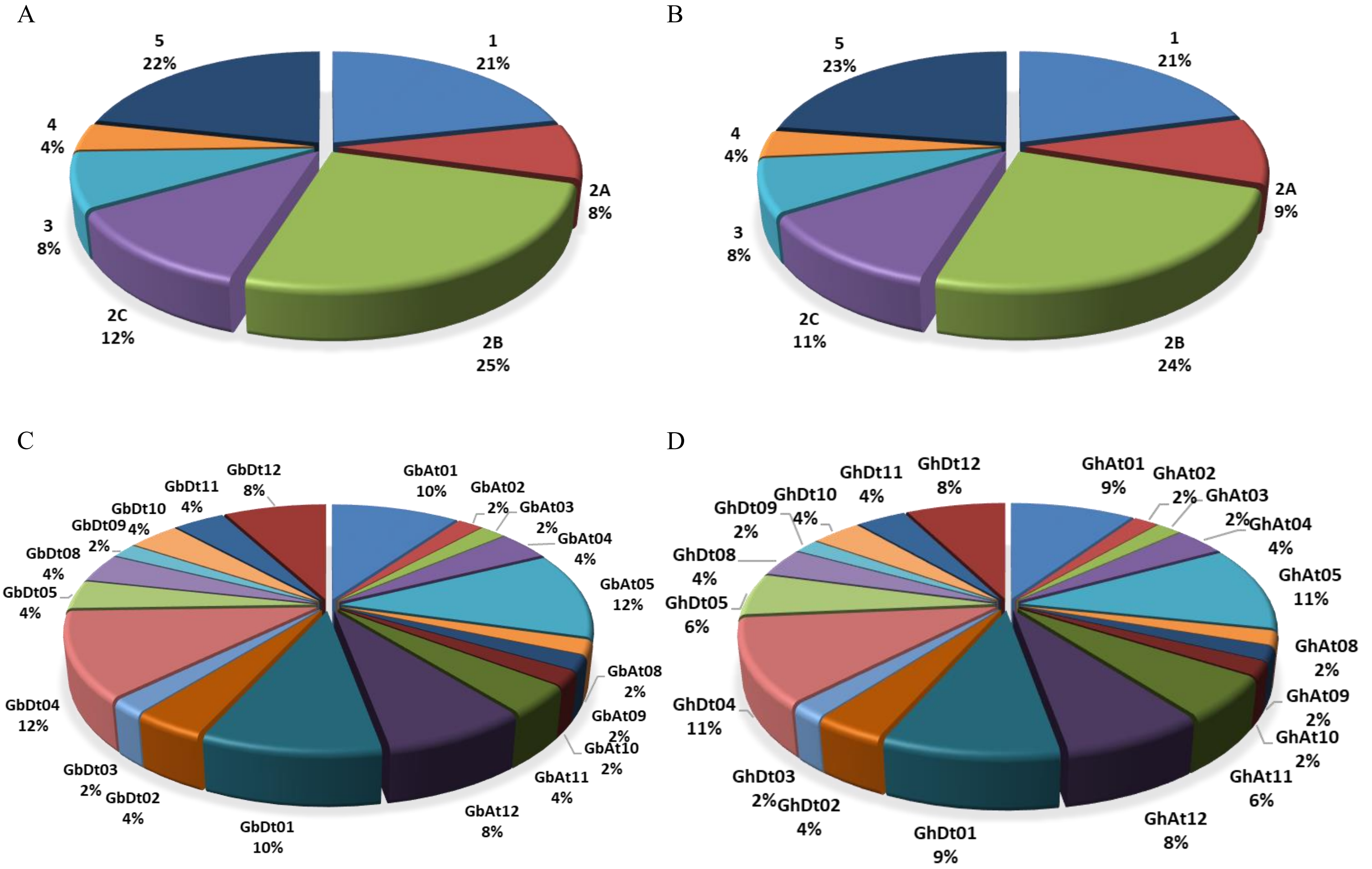

FIGURE S1 Percentage of KUP subfamilies (A-B) and their chromosomes localizations (C-D) in *G. barbadense* (A, C) and *G. hirsutum* (B, D).

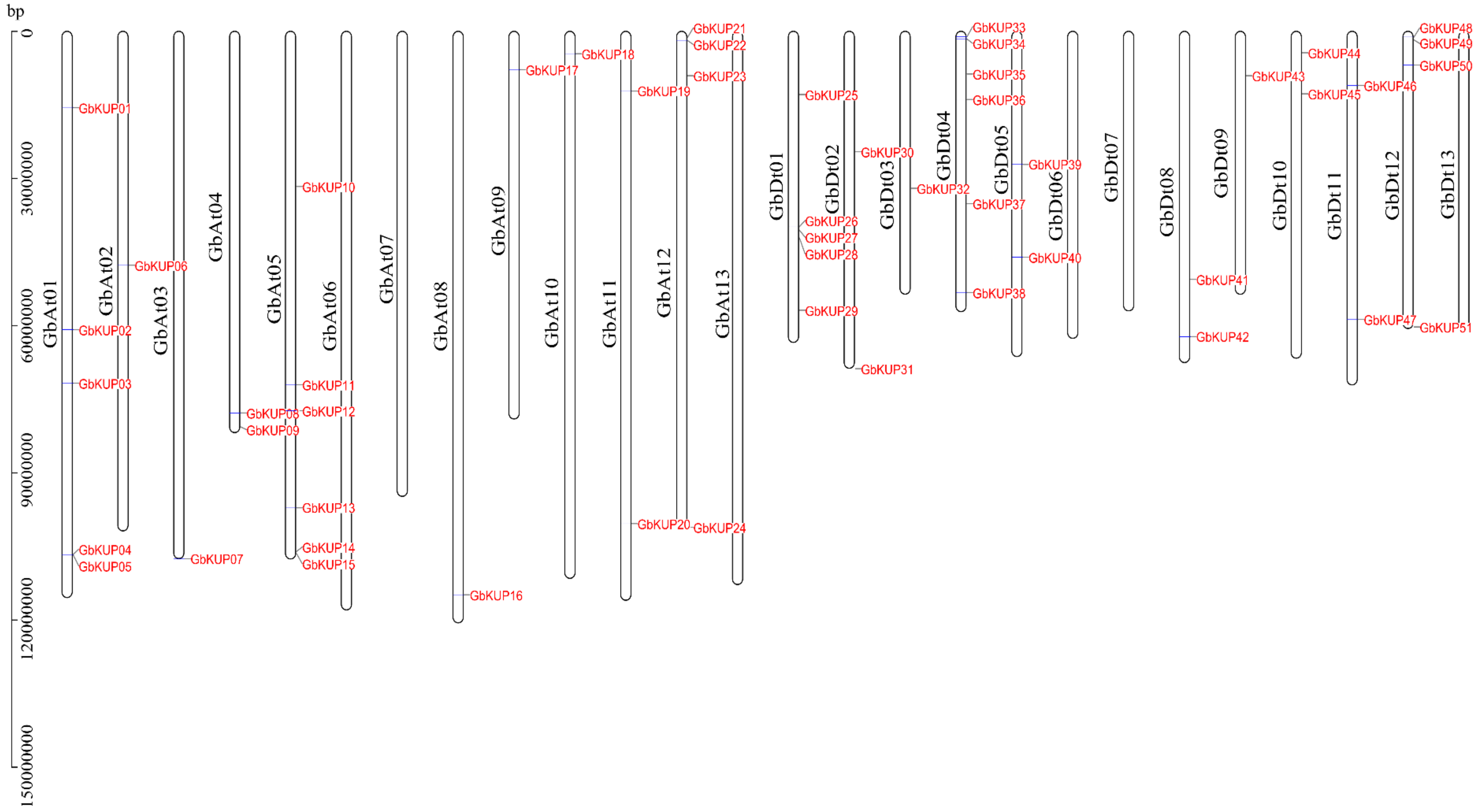

FIGURE S2 Chromosomal locations of GbKUPs across the *G. barbadense* chromosomes. The scale is base pair (bp).

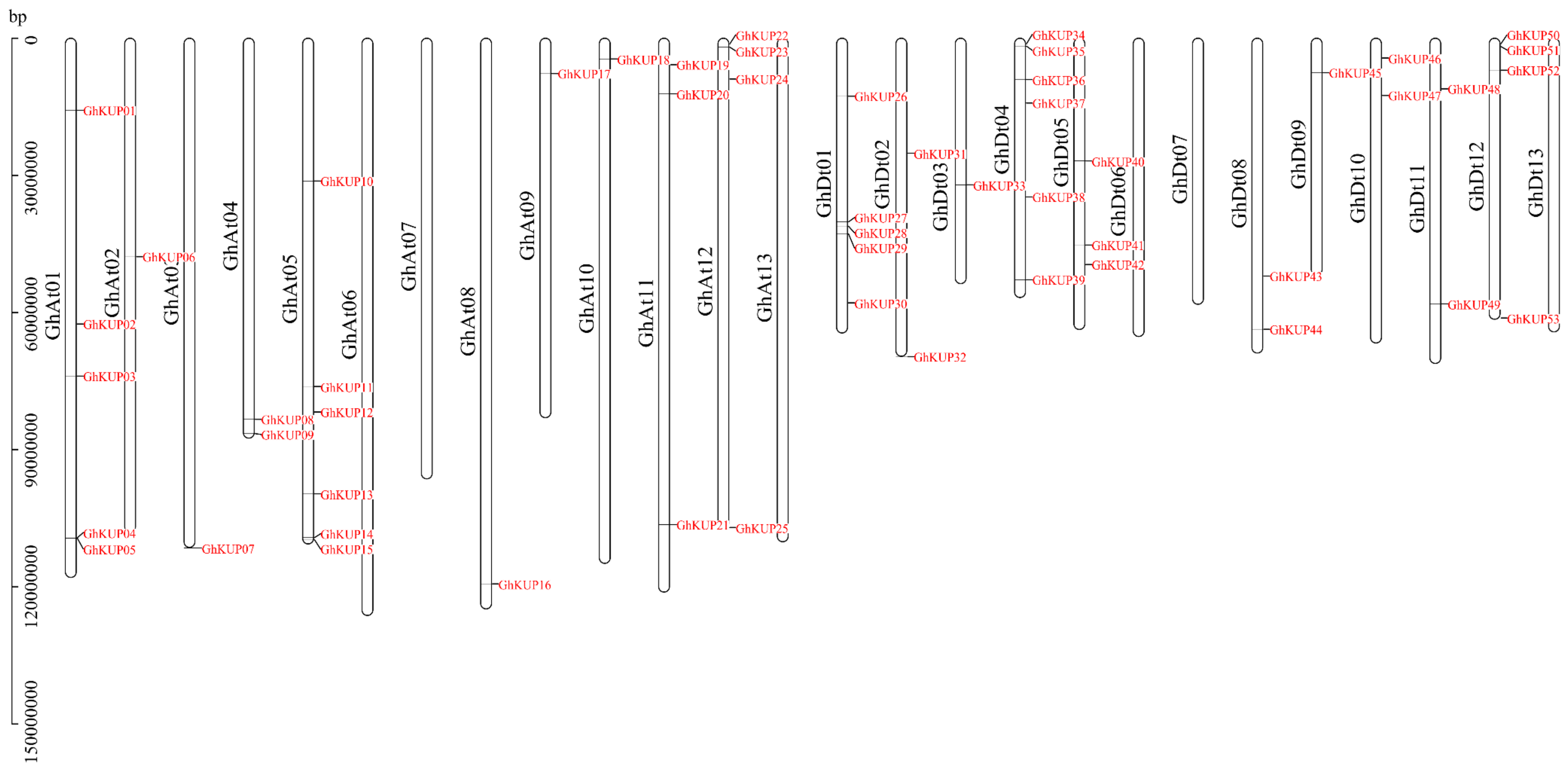

FIGURE S3 Chromosomal locations of GhKUPs across the *G.hirsutum* chromosomes. The scale is base pair (bp).
